# The role of the donor group and electron-accepting substitutions inserted in π-linkers in tuning the optoelectronic properties of D–π–A dye-sensitized solar cells: a DFT/TDDFT study†

**DOI:** 10.1039/d2ra00906d

**Published:** 2022-04-13

**Authors:** Hossein Roohi, Nafiseh Mohtamadifar

**Affiliations:** Computational Quantum Chemistry Laboratory, Department of Chemistry, Faculty of Science, University of Guilan Rasht Iran hroohi@guilan.ac.ir +98 131 3233262

## Abstract

The design of low-cost and high-efficiency sensitizers is one of the most important factors in the expansion of dye-sensitized solar cells (DSSCs). To obtain effective sensitizer dyes for applications in dye-sensitized solar cells, a series of metal-free organic dyes with the D–π–A–A arrangement and with different donor and acceptor groups have been designed by using computational methodologies based on density functional theory (DFT) and time-dependent density functional theory (TD-DFT). We have designed JK-POZ(1–3) and JK-PTZ(1–3) D–π–A–A organic dyes by modifying the donor and π-linker units of the JK-201 reference dye. Computational calculations of the structural, photochemical properties and electrochemical properties, as well as the key parameters related to the short-circuit current density and open-circuit voltage, including light-harvesting efficiency (LHE), singlet excited state lifetime (*τ*), reorganization energies (*λ*_total_), electronic injection-free energy (Δ*G*^inject^) and regeneration driving forces (Δ*G*^reg^) of dyes were calculated and analyzed. Moreover, charge transfer parameters, such as the amount of charge transfer (*q*^CT^), the charge transfer distance (*D*^CT^), and dipole moment changes (*μ*^CT^), were investigated. The results show that Δ*G*^reg^, *λ*_max_, *λ*_total_ and *τ* of JK-POZ-3 and JK-PTZ-3 dyes are superior to those of JK-201, indicating that novel JK-POZ-3 and JK-PTZ-3 dyes could be promising candidates for improving the efficiency of the DSSCs devices.

## Abstract 2

A series of metal-free organic dyes with the D–π–A–A arrangement and with different donor and acceptor groups have been designed theoretically.

## Graphical abstract

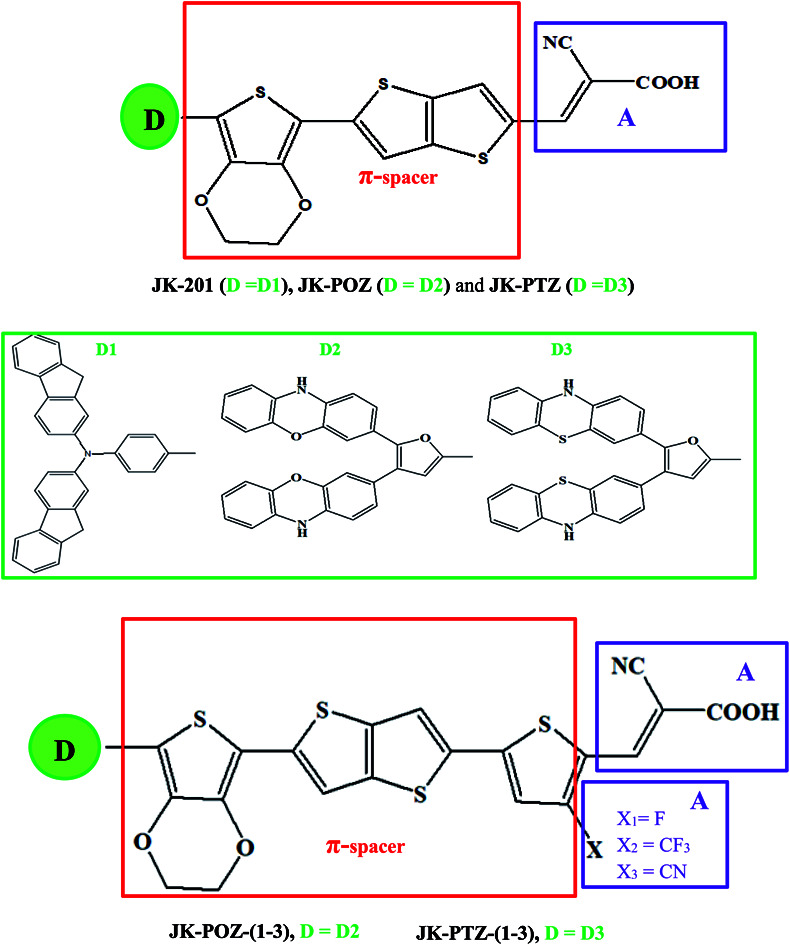

## 1. Introduction

In recent years, fossil fuels have been the main sources of energy, which present the hazards of the greenhouse effect, along with their chemical and nonrenewable properties. As such, there has been a necessity for clean renewable energy sources.^1,2^ Solar energy is one of the promising candidates for renewable energy sources, which has clean and unlimited energy resources.^3^ Therefore, the development and exploitation of solar energy have always been a challenge. In the past 10 years, polymer-based organic solar cells and perovskite solar cells have attained rapid development.^4^ Among the different designs of solar cells, dye-sensitized solar cells (DSSCs) have more evident advantages and are gradually becoming a part of human life.^5^ DSSCs were first reported by O'Regan and Grätzel in 1991.^6^ There has been significant development in the field of DSSCs, with the advantages of simple fabrication procedures, low-cost fabrication and high cell performance.^7,8^ The device structure of a typical DSSC has several important components: (1) nanoporous semiconductors TiO_2_, (2) dye sensitizer molecules, (3) counter electrodes and (4) redox electrolytes.^9^ The synthesized dyes are one of the most important components in DSSCs because they are the key components for converting solar energy into electricity.^10^ In the past decades, significant developments have been made to attain high efficiencies in DSSCs systems by introducing various dye molecules such as chlorophyll derivatives, porphyrins, ruthenium complexes, and metal-free organic materials.^11^

After the successful discovery by Grätzel *et al.*,^12^ DSSC devices based on N3 and N719, and Ru(ii)-photosensitizers have been extensively investigated.^13,14^ To date, the highest performances, with solar energy-to-electricity conversion efficiencies of 11% have been reported for the Ru sensitizers.^15–17^ However, the limited access, high cost of Ru-based dye and possible environmental problems could limit their wide application.^18,19^ More recently, metal-free organic sensitizers have been developed due to their advantages of low-cost preparation processes, higher extinction coefficients, environmental friendliness, higher structural flexibility easily tunable optical properties, ample availability and ease of purification.^20,21^

In general, metal-free organic sensitizers for DSSC applications consist of (D–π–A) molecular structures comprised of an electron donor (D) and an electron acceptor (A) bridged by a π-conjugated link. The D–π–A structure^22^ has a dipolar character based on the push–pull architecture, which is one of the most successful typologies of organic dyes.^23–25^ Recently, various donor groups have been reported in D–π–A dyes such as phthalocyanine, indoline,^26–28^ phenothiazine,^29–31^ coumarin,^32,33^ phenoxazine,^34,35^ carbazole^36–38^ and triphenylamine derivatives;^39,40^ they display high molar extinction coefficients due to facile intramolecular charge transfer.^30^ The cyanoacrylic acid structure has been applied generally as the A moiety in DSSC devices.

Among the different donors, phenothiazine (PTZ) and phenoxazine (POZ) donor moieties with unique electronic and optical properties are considered promising push–pull organic dye sensitizers for application in DSSCs.^41^ The PTZ and POZ rings exhibit nonplanar configuration, which could impede molecular aggregation.^41^ Recently, Han *et al.* designed and synthesized a PTZ-based organic dye (JY56), in which the alkoxy-phenyl- and hexyl-substituted PTZ group acts as the donor unit. The DSSC device based on dye JY56 gave efficiencies of 8.19%.^42^ The designed and synthesized dimer of organic dyes with different donor moieties of PTZ and POZ chromophores achieved the PCE of 5.87 and 6.40%.^43^ Wu *et al.* synthesized and applied a new organic dye containing the phenothiazine (TD2) donor with a power conversion efficiency of 5.40%.^44^ Three new phenothiazine-based organic dyes have been synthesized for DSSCs with PCEs of 3.78%, 4.41% and 2.48%, respectively.^45^ A series of novel metal-free organic dyes (POZ-2, POZ-3, POZ-4 and POZ-5) involving phenoxazine were synthesized as sensitizers for application in dye-sensitized solar cells (DSSCs).^46^ These dyes exhibited conversion efficiencies of 6.6%, 7.8%, 7.1% and 6.55%, respectively.^47^ A binary π-conjugation of a combination of the 3,4-ethylenedioxythiophene and thienothiophene entities was introduced into the conjugated spacer of organic dyes, which were prepared for dye-sensitized solar cells, and high conversion efficiencies were achieved. Wang *et al.* investigated the cell performance of sensitizers using an extension of a binary spacer of orderly conjugated 3,4-ethyldioxythiophene and thienothiophene units with a high power conversion efficiency of 9.8%.^48^ Grätzel, *et al.* designed and synthesized the novel dye sensitizer containing 3,4-ethylenedioxythiophene and thienothiophene in the bridging group.^49^

The optical properties of D–π–A dyes could be finely tuned by substituting appropriate D and A moieties, and π-bridges at suitable positions.^50–53^ The (*E*)-3-(5-(7-(4-(bis(9,9-dimethyl-9*H*-fluoren-2-yl)amino)phenyl)-2,3-dihydrothieno[3,4-*b*][1,4]dioxin-5-yl)thieno[3,2-*b*]thiophen-2-yl)-2-cyanoacrylic acid (JK-201)^54^ has been proposed as an effective donor–π bridge–acceptor sensitizer for DSSCs devices containing the bis-dimethylfluorenyl amino groups as donor units and cyanoacrylic acid as an acceptor moiety bridged by 3,4-ethylenedioxythiophene and thienothiophene with the maximum absorption centered at 481 nm, and a power conversion efficiency of 5.65%. The many significant efforts *via* theoretical computation have been taken into account for improving the device performance by modifying the sensitizers.^55^

**Fig. 1:**
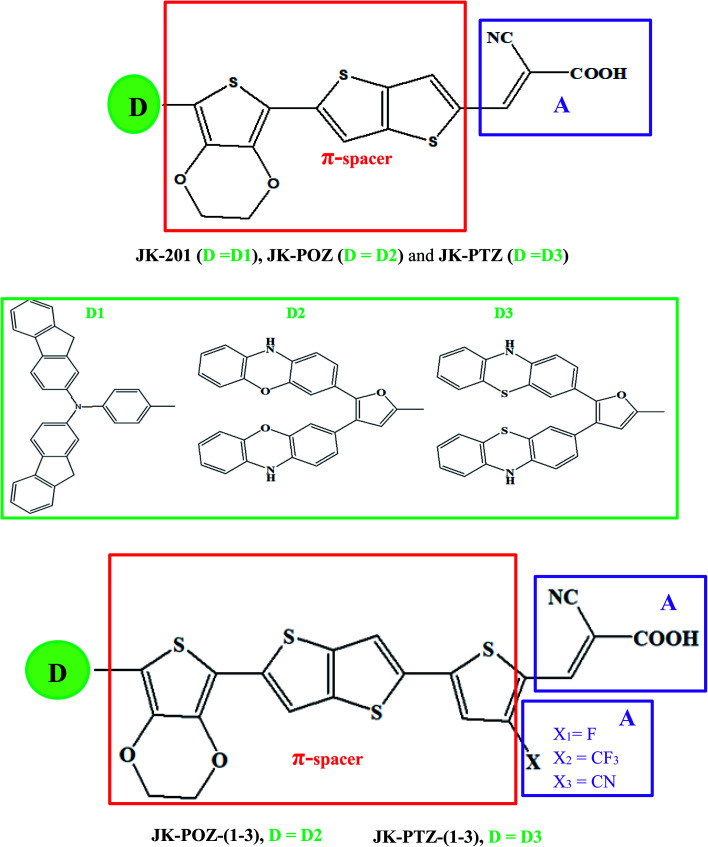
Modification strategy for the donor and π-conjugated bridge moieties of reference dye JK-201. Colored boxes indicate the constituents of the frame. Green (donor), red (π-conjugated bridge), violet (acceptor).

In this paper, we report the design of a series of organic dyes based on the experimentally studied JK-201 dye, illustrated in Fig. 1. The JK-201 dye was modified with changes in the original donor of the molecule and the selection of different auxiliary acceptors. Our designed D–π–A–A dyes contain phenothiazine and phenoxazine groups as donors, 3,4-ethylenedioxythiophene, thienothiophene, and thiophene as π-linkers, and electron-withdrawing substituents, and cyanoacrylic acid as the acceptor. The incorporated substituents at the *ortho*-position of the cyanoacetic acid can act as additional acceptor units to promote electron transfer to the anchoring group, utilizing the electron-withdrawing effect. We have computed the various optical and electrical properties of these designed dyes to realize the changes in the performance of DSSC by using density functional theory (DFT) and time-dependent DFT (TD-DFT) calculations.

## 2. Computational details

The optimized geometrical structures of the ground states, electronic properties, energy gaps and absorption spectra for the reference and designed dyes were calculated by using the density functional theory (DFT) and time-dependent density functional theory (TDDFT) methods, respectively. DFT calculations for all dyes were performed using the Gaussian 09 software.^51^ The ground-state geometry optimizations were performed at the B3LYP/6-31G(d,p) level of theory. Frequency analyses were carried out at the same theoretical level to ensure that the optimized geometries correspond to a local minimum on the potential energy surface. The vertical excitation energies (*E*_ext_, eV), maximum wavelengths (*λ*_max_, nm), oscillator strengths (*f*) and major electronic transitions (H = HOMO, L = LUMO) in THF solvent were calculated at the PCM/TD/LC-wPBE/6-31++G(d,p) level of theory.

Two main parameters determine the power conversion efficiency (PCE) of the solar cells: the short-circuit current density (*J*_SC_), and the open-circuit photo-voltage (*V*_OC_). The PCE can be defined by eqn (1):^56,57^1
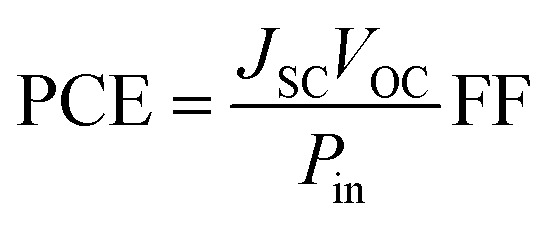
where FF is the fill factor and *P*_in_ is the incident solar power on the cell. The value of *J*_SC_ in DSSCs is determined as:^58^2
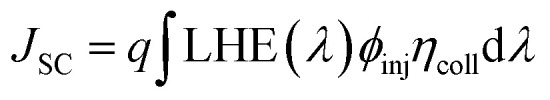
where *q* is the charge of the electron, LHE is light-harvesting efficiency, *η*_coll_ is the charge collection efficiency that can be considered as a constant for the same DSSC with only different sensitizers and *ϕ*_inj_ is the electron injection efficiency. The LHE of the dyes has to be as high as possible to maximize the *J*_SC_. LHE of investigated dyes is computed by using eqn (3):^59,60^

3 LHE = 1 − 10^−*f*^

where *f* is the oscillator strength corresponding to the wavelength *λ*_max_ of the dye.

The *ϕ*_*inj*_ is estimated by the free energy of electron injection (Δ*G*_inject_), which can be calculated as follows:^61^

4 Δ*G*_inject_ = *E*^dye∗^ − *E*_CB_

where, *E*^dye∗^ is the oxidation potential energy of the dye in the excited state and *E*_CB_ is the reduction potential of the conduction band (CB) of the TiO_2_ (−4.0 eV).^62^

The oxidation potential energy of the excited state can be estimated by

5 *E*^dye∗^ = *E*^dye^ − *E*_0–0_

where *E*^dye^ is the oxidation potential energy of the dye at the ground state (*E*^dye^ = −*E*_KS-HOMO_ (Kohn–Sham HOMO) and *E*_0–0_ is the electronic vertical transition energy corresponding to *λ*_max_.

The hole and the electron reorganization energy (*λ*_h_ and *λ*_e_) are given by the following equations:

6 *λ*_e_ = (*E*^−^_0_ − *E*^−^_−_) + (*E*^0^_−_ − *E*_0_)

7 *λ*_h_ = (*E*^+^_0_ − *E*^+^_+_) + (*E*^0^_+_ − *E*_0_)

The total reorganization energy (*λ*_total_) is calculated as

8 *λ*_total_ = *λ*_h_ + *λ*_e_

where *E*_0_, *E*^+^_0_ (*E*^−^_0_), *E*^+^_+_ (*E*^−^_−_) and *E*^0^_+_ (*E*^0^_−_) represent the neutral molecule optimized energy, the energy of the cation (anion) based on the neutral molecule geometry, the energy of the optimized cation (anion) structures and the energy of the neutral molecule calculated at the cationic (anionic) state, respectively. The cationic and anionic states of dyes were optimized at the same level of theory to calculate the total reorganization energies (*λ*_total_).

Moreover, the driving force regeneration Δ*G*_reg_ for the dye by oxidation potential energy was obtained using the following equation:^63^

9 Δ*G*_reg_ = *E*^dye^ − *E*_I^−^/I_3_^−^_

where *E*_I^−^/I_3_^−^_ is the redox potential energy of the redox electrolyte (−4.8 eV).^64^

The theoretical values of open-circuit voltage (*eV*_OC_) as a measure of electron-driving force have been calculated by using the following equation:^65,66^

10 *eV*_OC_ = *E*_LUMO_ − *E*_CB_

where *E*_LUMO_ is the energy of the LUMO and *E*_CB_ is the energy of the conduction band (CB) of the TiO_2_ semiconductor (∼−4.0 eV).

The molecular properties of the dyes were calculated at the B3LYP/6-31++G(d,p) level of theory in the gas phase. The ionization potential (IP) and electron affinity (EA) are the energies of the HOMO and LUMO orbitals,^67^ respectively.

11 IP = −*E*_HOMO_

12 EA = −*E*_LUMO_

Eqn (13) and (14) show an alternative method for calculating the IP and EA.^68^ In this part, an adiabatic approach was used to estimate the electron affinity (EA) and ionization potential (IP) energy. The molecular energies of the neutral and ionic species were calculated at the B3LYP/6-31++G(d,p) level of theory in the gas phase.

13 IP = *E*_cation_ − *E*_neutral_

14 EA = *E*_neutral_ − *E*_anion_

We also computed the chemical hardness (*η*), electrophilicity (*ω*), electron-donating power (*ω*^−^) and electron-accepting power (*ω*^+^) of dyes by using the following equations:^69,70^

15 *η* = 1/2(IP − EA)

16
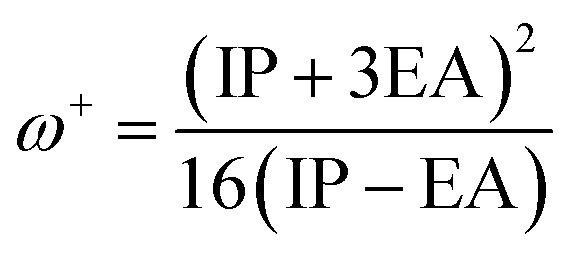
17
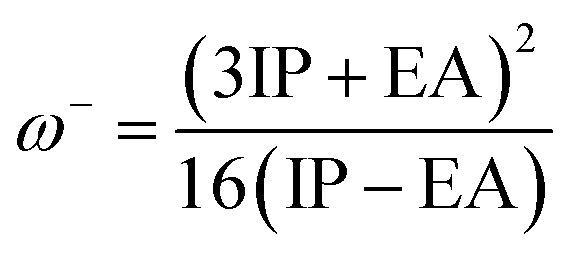
18
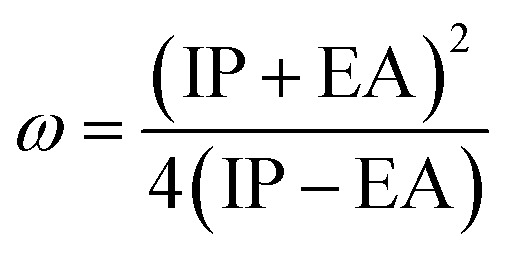


### 2.1. Validation of the computational method

To design novel organic dyes, the photophysical properties of the experimentally synthesized organic JK-201 dye were considered as a reference. The JK-201 dye structure consists of three parts: bis-(9,9-dimethyl-9*H*-fluoren-2-yl)-phenyl-amine (DMFA) as the donor (D) unit, 3,4-ethylenedioxythiophene and thienothiophene as the π-linker group and cyanoacrylic acid (CAA) as the acceptor (A). To find a suitable functional for the calculation of photophysical properties, the experimental UV-Vis spectra of the JK-201 dye were used as the reference data. Accordingly, TD-DFT calculations were performed using different functionals, namely mpwpw91, LC-wPBE, CAM-B_3_LYP and B3LYP with the 6-31++G(d,p) basis set in THF solution using the conductor-like polarizable continuum model (C-PCM).^71^Table 1 shows the computed *λ*_max_ with different functionals and experimental *λ*_max_ for JK-201 dye. The result calculated using the LC-wPBE functional is in good agreement with the experimentally obtained value. Therefore, we used the TD-LC-wPBE/6-31++G(d,p) level of theory for all dyes in THF to predict the optical properties of the novel dyes.

**Table 1:** The comparison of *λ* values obtained by experimental technique and computational calculations for the dye JK-201 in THF solution at the PCM/TD/DFT/6-31G++(d,p) level

	B3LYP	CAM-B3LYP	mpwpw91	LC-wPBE	Experimental
*λ*_max_	713.34	521.43	620.78	462.46	481

### 2.2. The structure of the designed dyes

**Fig. 2:**
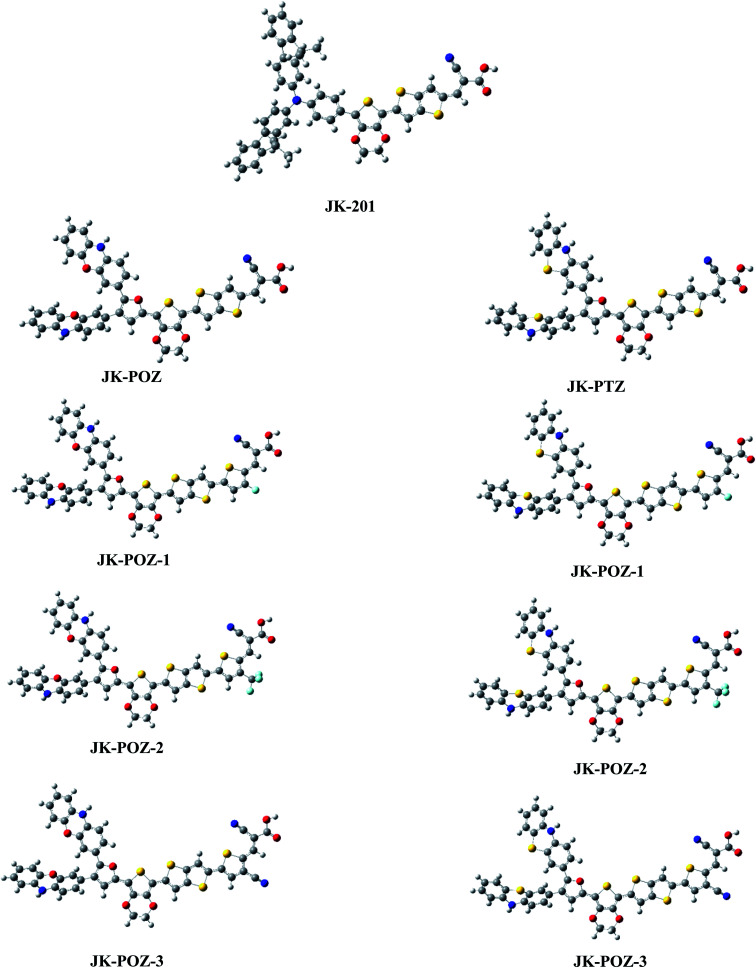
Optimized ground state geometries of the dyes JK-POZ-1–3, JK-PTZ-1–3 and reference dye JK-201.

Since the donor, linker, and acceptor groups play important roles in the absorption spectra, HOMO–LUMO energy gap and ICT properties, to develop efficient sensitizers, we have designed JK-POZ(1–3) and JK-PTZ(1–3) D–π–A–A organic dyes by modification of the donor, π-linker and electron-acceptor units of the JK-201 reference dye. In comparison with the JK-201 reference dye, in the JK-POZ and JK-PTZ dyes, only the donor groups were changed. In addition, in the JK-POZ(1–3) and JK-PTZ(1–3) dyes, thiophene units with three electron-withdrawing groups, namely –F, –CF_3_ and –CN, were added to the π-linker group of the JK-201 dye. The substituted sensitizers are named with suffixes 1-3, respectively. Therefore, eight new dyes were designed, namely JK-POZ, JK-PTZ, JK-POZ-1, JK-POZ-2, JK-POZ-3, JK-PTZ-1, JK-PTZ-2 and JK-PTZ-3. In these new dyes, in addition to the presence of substituents in the thiophene linked to the anchoring group, the length of the π-conjugate linker also increased as compared with the JK-201 reference dye. The B3LYP/6-31++G(d,p) optimized structures of all dyes are shown in Fig. 2.

### 2.3. Electronic properties

**Fig. 3:**
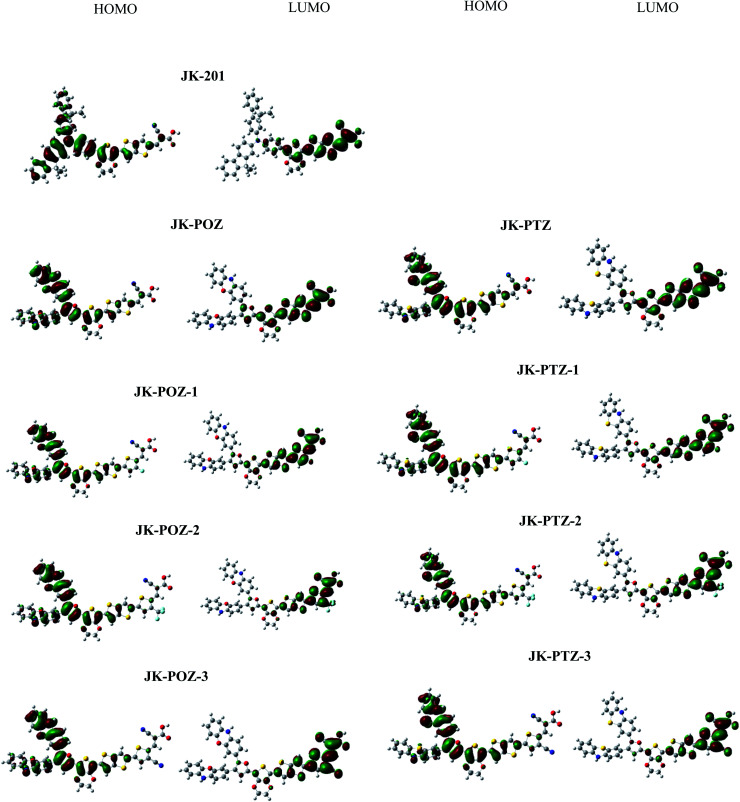
The HOMO and LUMO frontier molecular orbitals of the reference and designed dyes.

To understand the electronic connection between the LUMO of the dye and the TiO_2_ conduction band and the electron transfer features during light excitation, the optimized structure of dye molecules at the B3LYP/6-31++G(d,p) level of theory was used to analyze the frontier molecular orbitals (FMOs) of the dyes. In dye sensitizers, the intramolecular charge transfer (ICT) takes place from the electron donor part to the electron acceptor/anchoring moiety through the π-linkers. The ICT behavior in DSSCs devices can be understood from the structures of the highest occupied molecular orbital (HOMO) and lowest unoccupied molecular orbital (LUMO). The energy levels and the electronic distribution of FMOs of the dyes are the main factors in the electronic excitation properties of sensitizers.^72,73^ The anchoring carboxylate group present at the electron acceptor part increases dye adsorption on the basic TiO_2_ surface and directs the photo-induced electron injection. To promote the efficient photoelectron transfer from the dye to TiO_2_ and prevent the PCE-degrading back-electron transfer events, the LUMO orbital in dyes should be positioned near the TiO_2_ surface and the HOMO must be located far from it.^74^ The HOMO and LUMO orbitals are shown in Fig. 3. As can be seen, all dyes displayed the favored electronic state separation; the LUMO orbitals are mainly found on the anchoring group (–COOH) near the TiO_2_ surface and the HOMO are located on donor groups far from the TiO_2_ surface. The electron density of the HOMO of all dyes is predominantly located on the electron-donating moieties with delocalization onto the π-linker. The electronic density of the LUMO is mainly localized on the electron acceptor part with delocalization onto the π-linker. As can be seen, the π-linker in all dyes contributed to the HOMO as well as LUMO orbitals. This substantial HOMO–LUMO overlap at the π-bridge guarantees the efficient electron transfer from the donor to the acceptor and confirms that an ICT occurs from the electron-donor group to the electron-acceptor moiety through the π-linker. The anchoring group (–COOH) of all dyes has a significant contribution to the LUMO orbital, which could lead to a strong electronic injection into the conduction band (CB) of the TiO_2_ electrode, subsequently increasing the short-circuit current density *J*_SC_.

**Fig. 4:**
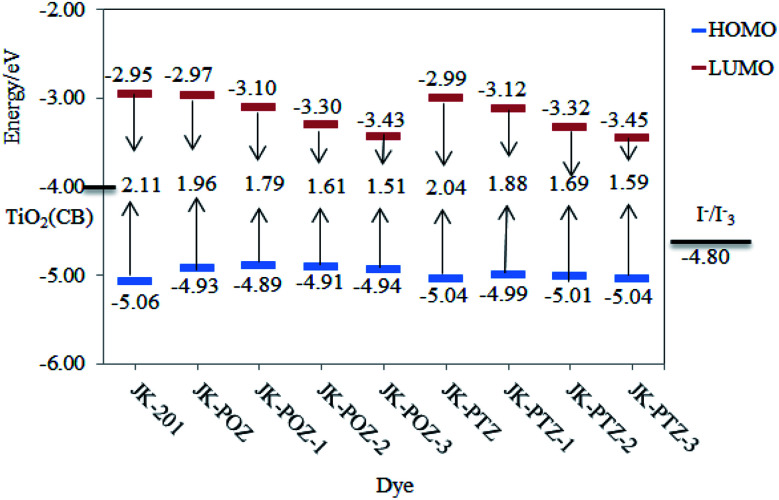
The HOMO and LUMO energy levels for JK-POZ-1–3, JK-PTZ-1–3 and JK-201 dyes.

The HOMO and LUMO energy values for JK-201 and JK-POZ, JK-PTZ, JK-POZ-1, JK-POZ-2, JK-POZ-3, JK-PTZ-1, JK-PTZ-2 and JK-PTZ-3 dyes are provided in Table 2. Thermodynamically, for spontaneous electron injection from the dye excited state to TiO_2_ conduction in DSSC systems, the LUMO energy value of the dye must be greater than the conductance band (CB) of TiO_2_ (−4.0 eV).^75^ Besides, for the spontaneous regeneration of electrons, the HOMO energy level of dye must be more negative than the redox potential of the I^−^/I_3_^−^ electrolyte (−4.80 eV).^76^ The HOMO and LUMO energy levels for experimentally synthesized dye JK-201 and theoretically designed dyes at the B3LYP/6-31++G(d,p) level in the gas phase are displayed in Fig. 4. The LUMO energies of all dyes (ranging from −3.45 to −2.95 eV) are much higher than the conduction band of TiO_2_ (−4.0 eV) and simultaneously, the HOMO energies of sensitizers (ranging from −5.06 to −4.89 eV) are lower than the redox potential energy of the electrolyte (−4.80 eV). Therefore, the effective injection of excited electrons and, subsequently, the efficient regeneration of the oxidized dyes are energetically favorable. This indicates that the designed dyes are suitable for DSSC systems.

**Table 2:** The HOMO and LUMO FMO energies and energy gaps (in eV) of dyes at the B3LYP/6-31++G(d,p) level of theory in the gas phase. The TD-LC-wPBE/6-31++G(d,p) results in THF solvent are given in parentheses

Dye	*E*_HOMO_	*E*_LUMO_	*E*_g_
JK-201	−5.06(−5.05)	−2.95(−3.09)	2.11(1.96)
JK-POZ	−4.93(−4.92)	−2.97(−3.10)	1.96(1.82)
JK-POZ-1	−4.89(−4.88)	−3.10(−3.21)	1.79(1.67)
JK-POZ-2	−4.91(−4.89)	−3.30(−3.40)	1.61(1.49)
JK-POZ-3	−4.94(−4.89)	−3.43(−3.51)	1.51(1.38)
JK-PTZ	−5.04(−5.07)	−2.99(−3.12)	2.04(1.95)
JK-PTZ-1	−4.99(−5.01)	−3.12(−3.22)	1.88(1.79)
JK-PTZ-2	−5.01(−5.02)	−3.32(−3.41)	1.69(1.61)
JK-PTZ-3	−5.05(−5.03)	−3.45(−3.52)	1.59(1.51)

Interestingly, the HOMO level is closely relevant to the electron-donors unit, while the LUMO energy level is mainly affected by the electron-drawing group.^77^ From Table 2, the HOMO and LUMO energy levels of the unsubstituted JK-POZ and JK-PTZ dyes are −4.93, −5.04 eV and −2.97, −2.99 eV, respectively. In comparison with the JK-201 reference dye, the HOMO energy level of JK-POZ and JK-PTZ dyes is significantly up-shifted and the LUMO energy level is slightly down-shifted. However, insertion of the substituted thiophene ring into the π-conjugated bridge of these dyes leads to a significant change in both the HOMO and LUMO energy levels. For both JK-PTZ and JK-POZ-based substituted dyes, the LUMO energy decreases in the following order: 1 < 2 < 3, as compared with the main dyes. Consequently, the calculated LUMO energy level gradually decreases with the incorporation of the electron-withdrawing substituents. The JK-PTZ-3 and JK-POZ-3 dyes having the CN group show the lowest LUMO energy levels among the investigated dyes. These results confirm that substituting the primitive JK-PTZ and JK-POZ dyes with electron-withdrawing moieties can lead to a decrease in the LUMO energy levels of the sensitizers, in good agreement with the results of the effect of fluorine substituents in the π-spacer on the FMOs energies of dyes;^78,79^ both HOMO and LUMO energies were decreased with the substitution of the F atoms. As can be seen in Fig. 4, although the HOMO energy of all dyes is greater than the JK-201 reference dye, the presence of substituted thiophene has no significant effect on the HOMO levels. The increase in HOMO energy can facilitate the excitation of electrons to LUMO levels.

The energy gap (*E*_g_ = *E*_LUMO_ − *E*_HOMO_) is the main factor in the efficiency of the solar cell. The low value of *E*_gap_ leads to a strong absorption band in the electronic spectra and better intramolecular charge transfer (ICT). The energy gaps of the dyes are summarized in Table 2. The *E*_gap_ of the experimentally synthesized dye JK-201 and theoretically designed dyes decrease in the order of JK-201 > JK-PTZ > JK-POZ > JK-PTZ-1 > JK-POZ-1 > JK-PTZ-2 > JK-POZ-2 > JK-PTZ-3 > JK-POZ-3. Among the substituted forms of JK-POZ and JK-PTZ-based dyes, the energy gap of JK-POZ-3 and JK-PTZ-3 is the lowest (1.51 eV and 1.59 eV) due to the presence of the electron acceptor CN group, implying that the JK-POZ-3 and JK-PTZ-3 dyes would display the best optical properties among the studied dyes. The observed trend can be explained by the order of the decrease in the LUMO energy level. Thus, the band gap energy of both JK-POZ and JK-PTZ-based dyes strongly depends on the LUMO energy. The decrease in the band gap energy upon the substitution of electron-accepting groups is in good agreement with the results obtained in the study of the effects of fluorine substituents in the π-spacer on the energy gaps of the dyes^74,75^ and the effects of electron-withdrawing groups (–CF_3_, –COCl, –F and –NO_2_) attached at the donor and the acceptor units on the FMO energies and energy gaps of dyes.^80^ Huang *et al.*^81^ investigated the effects of CF_3_ group substitution on the *ortho* and *para*-benzene positions in the acceptor section. They concluded that with the substitution of the CF_3_ group, the LUMO energy values and the gap energy decreased, in good agreement with the results of our work.

### 2.4. Chemical reactivity parameters

The chemical reactivity parameters consist of adiabatic and vertical electron affinity (EA_a_/EA_v_), adiabatic and vertical ionization potential (IP_a_/IP_v_), chemical hardness (*η*), electrophilicity index (*ω*), electron-donating power (*ω*^−^), and electron-accepting power (*ω*^+^), which were calculated. The obtained values are listed in Table 3. The ionization potential can be defined as the energy change on adding holes or extracting electrons in the molecule, and electron affinity can be seen as the energy change on absorbing electrons or extracting holes in the molecule.^64^ The sensitizer dye plays a dual role in DSSCs since it injects the excited electron into TiO_2_ and obtains an electron from the electrolyte to fill the hole. By studying the electron affinities and ionization potentials, the related information about the gain and loss of electrons or holes in dyes can be obtained.

**Table 3:** The chemical reactivity of the original and designed dyes including the adiabatic and vertical ionization potentials, adiabatic and vertical electron affinities, chemical hardness, electrophilicity index, electron-donating and electron-withdrawing powers (in eV) calculated at the B3LYP/6-31++G(d,p) level of theory

Dye	IP_v_	EA_v_	IP_a_	EA_a_	*η*	*ω*	*ω* ^+^	*ω* ^−^
JK-201	5.06	2.95	5.85	2.05	1.06	7.60	5.73	9.74
JK-POZ	4.93	2.97	5.68	2.03	0.98	7.94	6.09	10.04
JK-POZ-1	4.89	3.10	5.62	2.20	0.90	8.90	7.01	11.01
JK-POZ-2	4.91	3.30	5.64	2.37	0.80	10.48	8.53	12.63
JK-POZ-3	4.89	3.43	5.67	2.48	0.75	11.62	9.62	13.81
JK-PTZ	5.04	2.99	5.78	2.06	1.02	7.88	6.00	10.02
JK-PTZ-1	4.99	3.12	5.71	2.22	0.94	8.77	6.86	10.92
JK-PTZ-2	5.01	3.32	5.73	2.39	0.85	10.27	8.29	12.46
JK-PTZ-3	5.04	3.45	5.76	2.50	0.80	11.33	9.31	13.55

Calculated IPs and EAs of the reference and designed dyes are listed in Table 3. As can be seen from this table, the IP of designed dyes is less than that of the JK-201 reference dye, indicating that the structural modifications could result in the dyes losing electrons more easily, thereby giving the designed dyes better photoelectrical properties. Besides, the EAs of the designed dyes are greater as compared to the JK-201 reference dye, indicating that the tendency of designed dyes to accept electrons is greater as compared to the reference dye. However, the calculated EA values of the reference and designed dyes show a great difference between them and the order of EA is JK-PTZ-3 > JK-POZ-3 > JK-PTZ-2 > JK-POZ-2 > JK-PTZ-1 > JK-POZ-1 > JK-PTZ > JK-POZ > JK-201. The results imply that the JK-PTZ-3 and JK-POZ-3 dyes represent the best electron-accepting ability among the investigated dyes.

The calculated *η*, *ω*, *ω*^+^ and *ω*^−^ chemical reactivity parameters of the original and designed dyes are shown in (Table 3). To increase the charge transfer and separation, the chemical hardness as a measure of the resistance of the dyes to intramolecular charge transfer should be a small value. From Table 3, the *η* values of all investigated dyes were found to be less than the JK-201 reference dye and are in the following decreasing order: JK-201 > JK-PTZ > JK-POZ > JK-PTZ-1 > JK-POZ-1 > JK-PTZ-2 > JK-POZ-2 > JK-PTZ-3 > JK-POZ-3. As shown in Table 3, the JK-POZ-3 and JK-PTZ-3 dyes have the lowest chemical hardness among the investigated dyes.

The stabilization energies of molecular structures may be measured by the value of *ω*; the highest *ω*^+^ value indicates the highest electron-accepting ability and, therefore, higher *ω* and *ω*^+^ are preferable.^82^Table 3 shows that the order of increasing *ω* and *ω*^+^ for the original and designed dyes is JK-POZ-3 > JK-PTZ-3 > JK-POZ-2 > JK-PTZ-2 > JK-POZ-1 > JK-PTZ-1 > JK-POZ > JK-PTZ > JK-201. Therefore, the designed JK-POZ-3 and JK-PTZ-3 dyes showed the highest electron-accepting power and stabilization energy among the reference and other dyes. Moreover, the calculated electron-donating powers of the reference and designed dyes (*ω*^−^) display the same trend of electrophilicity and electron-accepting power. Therefore, according to the above parameters, the JK-POZ-3 and JK-PTZ-3 dyes have the best stabilization energy, the best electron-accepting ability, and the lowest chemical hardness, and in turn, greater short-circuit current density, and better power conversion efficiency (PCE).

### 2.5. Natural bond orbital (NBO) analysis

The main aspect of population analysis is to provide a picture of the charge distribution and electron transfer from the D (filled) orbital of a subsystem to the A (vacant) orbital of other subsystems in D–π–A structures.^83^ To gain insight on the charge population of the investigated compounds, NBO population analysis was performed based on the optimized geometries of the ground state. The calculated NBO results at the B3LYP/6-31++G(d,p) level are tabulated in Table 4. In all dyes, the donor moiety contains the positive value of the total NBO charge, which demonstrated that they act as an effective electron-donor group. Contrarily, the negative NBO charges in the auxiliary acceptor and acceptor/anchoring groups reveal which electrons are trapped in the electron-acceptor moiety. The positive value of the charge for π-conjugated linkers demonstrates that they may not trap the electron and act as a transporter for the electron-transfer mechanism from D to A units. In the case of JK-POZ and JK-PTZ dyes, the D and the π-bridge have positive charge values and the A moiety has a negative charge. Among all the investigated dyes, the highest total NBO charge values of the D, π-linker and A moieties were observed in JK-POZ and JK-PTZ (Table 4). These results prove that the electrons are probably transferred efficiently from the D and A moieties through the π-conjugated linker in all investigated dyes, which leads to a proficient intramolecular charge separation state between D and A units and, finally, injection into the conduction band of TiO_2_.

**Table 4:** The natural bond orbital charge (*e*) of the fragments of dyes in the ground state at the B3LYP/6-31++G(d,p) level of theory

Dye	Donor	Linker	Acceptor
JK-201	0.0528	0.1347	−0.1759
JK-POZ	0.0941	0.1237	−0.1792
JK-POZ-1	0.0745	0.0649	−0.1229
JK-POZ-2	0.0862	0.0877	−0.1401
JK-POZ-3	0.0862	0.0910	−0.0928
JK-PTZ	0.0636	0.1348	−0.1766
JK-PTZ-1	0.0630	0.0680	−0.1202
JK-PTZ-2	0.0653	0.0906	−0.1370
JK-PTZ-3	0.0688	0.0940	−0.0987

### 2.6. Absorption properties

**Fig. 5:**
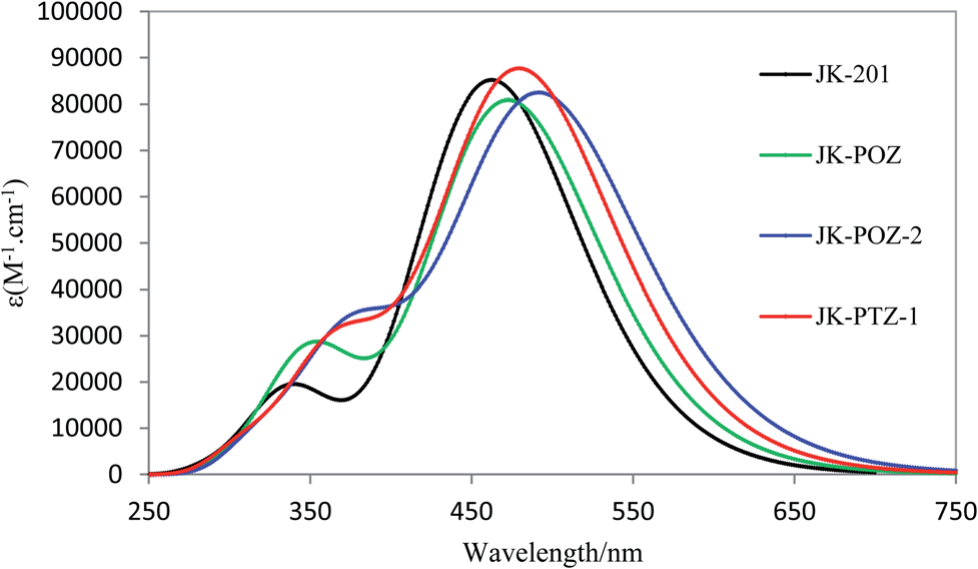
Calculated absorption spectra of the reference and most important designed dyes at the PCM/LC-wPBE/6-31++G(d,p) level of theory in THF solvent.

Although energy gaps are the main factors used to evaluate the optical properties, UV-Vis absorption spectra provide more reliable data to evaluate how the sensitizers will affect the efficiency of the solar cells. The UV-Vis absorption spectra of the sensitizers were computed by using the C-PCM model in THF solution at the TD-DFT/LC-wPBE/6-31++G(d,p) level of theory. The calculated vertical excitation energy (*E*_ext_), transition energy, oscillator strength (*f*), and the light-harvesting efficiency (LHE) of all sensitized dyes are given in Table 5. The absorption spectra of the studied dyes obtained at the LC-wPBE/6-31++G(d,p) level are shown in Fig. 5 and S1 (ESI†). Spectra of the reference dye and designed dyes are similar and show two distinct absorption bands in the ultraviolet and visible regions. It can be seen that the *λ*_max_ range for the dyes varies from 462.46 nm to 493.26 nm due to the broad intramolecular charge transfer (ICT) band. From Table 5, the maxima absorption peaks corresponding to JK-201, JK-POZ, JK-POZ-1, JK-POZ-2, JK-POZ-3, JK-PTZ, JK-PTZ-1, JK-PTZ-2, JK-PTZ-2, and JK-PTZ-3 are 462.5 nm, 473.0, 483.1, 493.3, 492.89, 469.2, 480.6, 490.1 and 489.8 nm in THF solvent, respectively. As can be seen, *λ*_max_ increases on going from experimentally synthesized JK-201 dye to designed dyes as the π-conjugation linker group is extended. The JK-201 dye had the lowest absorption wavelength value of 462.46 nm, while dyes JK-POZ-2, JK-POZ-3, JK-PTZ-2, and JK-PTZ-3 had the greatest *λ*_max_, in good agreement with the trend observed for *E*_gap_. The computed *λ*_max_ of the reference dye was found to be 462.5 nm, which is in very good agreement with the experimental value of 481 nm.

**Table 5:** Computed vertical excitation energies (*E*_ext_, eV), maximum wavelengths (*λ*_max_, nm), oscillator strengths (*f*) and major electronic transitions (H = HOMO, L = LUMO) calculated at the PCM/LC-wPBE/6-31++G(d,p) level of theory in THF solvent

Dye	Transitions	*E*_ext_	*λ*_max_	*f*	Major electronic transitions		
JK-201	S_0_–S_1_	2.68	462.4 (481)^a^	2.10	H−1 → L(41%)	H−4 → L(2%)	H → L+1(5%)
					H → L(41%)	H−3 → L(3%)	
JK-POZ	S_0_–S_1_	2.621	472.96	1.99	H−1 → L(5%)	H−3 → L(7%)	H → L+1(6%)
					H−2 → L(33%)	H−3 → L+1(2%)	
					H → L(39%)		
JK-POZ-1	S_0_–S_1_	2.566	483.11	2.15	H−1 → L(13%)	H−2 → L(22%)	H → L+1(8%)
					H−3 → L (12%)	H → L(31%)	
JK-POZ-2	S_0_–S_1_	2.515	492.89	1.94	H−1 → L(12%)	H−3 → L (14%)	H → L+1(9%)
					H → L(29%)	H−2 → L(21%)	
JK-POZ-3	S_0_–S_1_	2.514	493.26	2.04	H−1 → L(10%)	H−3 → L (14%)	H → L+1(10%)
					H → L(27%)	H−2 → L(21%)	H−5 → L (3%)
						H−2 → L+1(2%)	
JK-PTZ	S_0_–S_1_	2.643	469.15	1.99	H−1 → L(10%)	H−2 → L(18%)	H → L+1(6%)
					H → L(48%)	H−3 → L(7%)	
JK-PTZ-1	S_0_–S_1_	2.580	480.63	2.15	H−1 → L(21%)	H−2 → L(7%)	H → L+1(9%)
					H → L(40%)	H−3 → L (11%)	
JK-PTZ-2	S_0_–S_1_	2.530	490.11	2.00	H−1 → L(20%)	H−2 → L(7%)	H → L+1(10%)
					H → L(37%)	H−3 → L (12%)	H−5 → L(3%)
JK-PTZ-3	S_0_–S_1_	2.532	489.76	1.90	H−1 → L(18%)	H−2 → L(8%)	H → L+1(11%)
					H → L(34%)	H−3 → L (13%)	H−5 → L(3%)

a Experimental values in parentheses from S. Paek, H. Choi, H. Choi, C. W. Lee, M. S. Kang, K. Song, M. S. Nazeeruddin and J. Ko, Molecular engineering of efficient organic sensitizers incorporating a binary π-conjugated linker unit for dye-sensitized solar cells, *The Journal of Physical Chemistry C*, 2010, **114**(34), 14646–14653.

As seen in Table 5, the comparison of *λ*_max_ values revealed redshifts of 10.5, 20.65, 30.43, 30.80, 6.69, 18.17, 27.65, and 27.30 nm in the extended π-conjugation substituted dyes, compared with the JK-201 dye, so that the greatest redshifts in *λ*_max_ correspond to the JK-POZ-2, JK-POZ-3, JK-PTZ-2 and JK-PTZ-3 dyes. The red-shifted absorption spectra suggest the possible advantage of the light-harvesting ability, which is favorable for further increasing the efficiency of the corresponding solar cells. As a result, the judicious modification of the JK-201 dye is an effective strategy for improving the absorption spectrum of the JK-201 dye.

### 2.7. Intramolecular charge transfer

To quantify the amount of ICT in the investigated dyes,^84,85^ the amount of transferred charge (*q*^CT^), the distance of charge transfer (*D*^CT^), and the exciton binding energy (*E*_b_) were calculated. The “Δ” index represents the difference between *D*^CT^ and *H* (Δ = *D*^CT^ − *H*), which can be applied to quantify the through-space character associated with a CT excitation. The *H* value is defined as half the sum of two centroid axes along with the electron transfer D–A direction (*x*-axis). The change in the dipole moment upon S_0_ → S_1_ transitions is caused by charge rearrangement or intramolecular charge transfer. This variation in the dipole moment of the dye can be defined as *μ*^CT^ = *D*^CT^*q*^CT^. The intramolecular charge transfer indexes were computed at the B3LYP/6-31++G(d,p) by the Multiwfn code.^86^ These parameters are collected in Table 6.

**Table 6:** Calculated charge transfer parameters *q*^CT^ (e^−^), *D*^CT^ (Å), *H* (Å), Δ (Å) and *μ* (D) for the studied dyes in the gas phase at the B3LYP/6-31++G(d,p) level of theory

Dye	*q* ^CT^	*D* ^CT^	*H*	Δ	*μ* ^CT^
JK-201	0.986(−0.990)^a^	6.740	7.012	3.059	31.9
JK-POZ	0.971(−0.965)	6.718	6.703	2.470	31.3
JK-POZ-1	1.022(−1.029)	7.660	7.571	2.711	37.6
JK-POZ-2	1.067(−1.068)	7.772	7.698	2.871	39.8
JK-POZ-3	1.072(−1.074)	8.006	7.762	2.802	41.2
JK-PTZ	0.933(−0.933)	6.518	6.672	2.437	29.2
JK-PTZ-1	0.973(−0.976)	7.513	7.482	2.854	35.2
JK-PTZ-2	1.026(−1.030)	7.502	7.658	2.930	37.0
JK-PTZ-3	1.039(−1.042)	7.852	7.680	2.912	39.2

a Charges associated with the positive and negative density regions.

The calculated results of *D*^CT^ and *q*^CT^ values of the reference and designed dyes are given in Table 6. Under the first excitation, the *D*^CT^ values are in the order of JK-POZ-3 > JK-PTZ-3 > JK-POZ-2 > JK-POZ-1 > JK-PTZ-1 > JK-PTZ-2 > JK-201 > JK-POZ > JK-PTZ. It shows that the substituted JK-POZ(1–3) and JK-PTZ(1–3) dyes have larger *D*^CT^ in comparison to the reference dye molecule (JK-201). The larger the *D*^CT^, the lower is the overlap of the electron–hole distribution and the greater the efficiency of the DSSC. This may be related to the stronger ability of the electron-withdrawing substituents. Besides, the H values increase in the following order: JK-POZ-3 > JK-POZ-2 > JK-PTZ-3 > JK-PTZ-2 > JK-POZ-1 > JK-PTZ-1 > JK-201 > JK-POZ > JK-PTZ. This indicates that the H values for JK-POZ(1–3) and JK-PTZ(1–3) dyes are greater than other ones. The large separation between the density increment and depletion regions is related to the larger value of *Δ*.^87^ From Table 6, it can be found that the *Δ* values for the substituted JK-POZ(1–3) and JK-PTZ(1–3) dyes are greater than the main JK-POZ and JK-PTZ dyes. The larger the *Δ* value is, the larger the charge separation in the ICT process.

The transferred charge *q*^CT^ is the magnitude of the integral of (*ρ*^+^) and (*ρ*^−^) over the whole space.^88^ Two functions *ρ*^+^ and *ρ*^−^ can be given to define the increase and decrease of the density owing to the electron transition. It is important to correctly recognize the physical meaning of this quantity. The *q*^CT^ only corresponds to the total amount of charge distribution perturbed during electron excitation; it does not correspond to the net charge transfer from one fragment to another fragment (*e.g.* from donor group to acceptor group). The *q*^CT^ values of dyes increase in the following order: JK-POZ-3 > JK-POZ-2 > JK-PTZ-3 > JK-PTZ-2 > JK-POZ-1 > JK-201 > JK-PTZ-1 > JK-POZ > JK-PTZ. Most of the dyes (except JK-PTZ-1, JK-POZ, and JK-PTZ) possessed *q*^CT^ values larger than the parent dye (JK-201). The *D*^CT^ and *q*^CT^ values are more substantial for JK-POZ(2–3) and JK-POZ(2–3), implying that the ICT is more likely to occur in the case of these dyes as compared with the other original and designed dyes.

Furthermore, *μ*^CT^, is the main factor in estimating the ICT properties of dyes. The data in Table 6 show that its value increases in the order of JK-POZ-3 > JK-POZ-2 > JK-PTZ-3 > JK-POZ-1 > JK-PTZ-2 > JK-PTZ-1 > JK-201 > JK-POZ > JK-PTZ. As can be seen, *μ*^CT^ values for both JK-POZ and JK-PTZ-based substituted dyes are greater as compared to unsubstituted and reference JK-201 dyes, indicating that electron-withdrawing substituents in the thiophene ring have a large effect on the net negative charges of the acceptor and anchoring groups. The better charge transfer indexes such as *q*^CT^, *D*^CT^, *H*^CT^ and *μ*^CT^ for substituted designed dyes (JK-POZ(1–3) and JK-PTZ(1–3)) indicate that these dyes may facilitate the ICT process and in turn, improve the efficiency of the DSSCs.

**Fig. 6:**
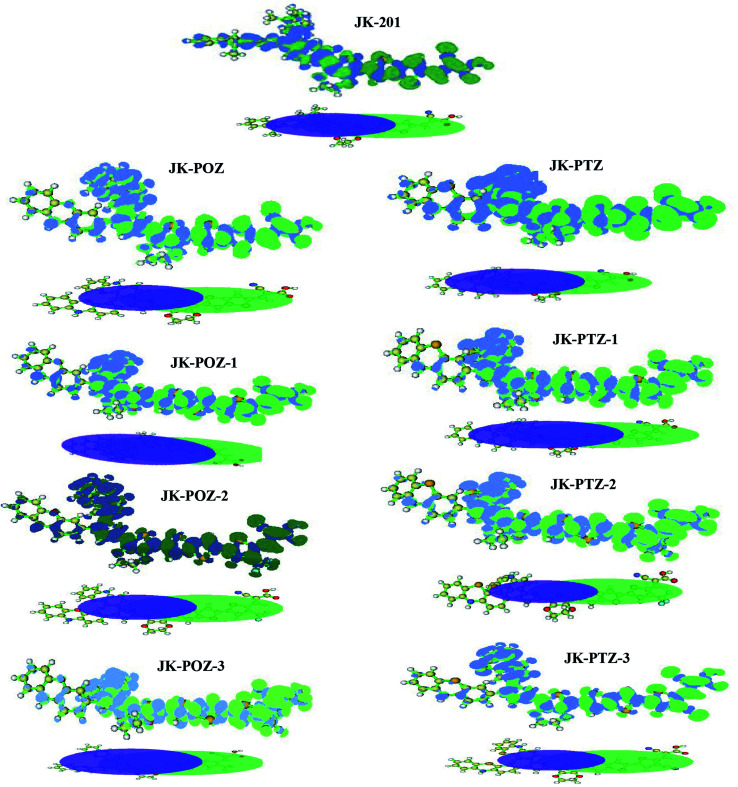
The calculated charge density differences between the ground and excited states of the reference and designed dyes. Green and blue regions correspond to positive and negative regions, respectively; they represent an increase and decrease in electron density due to the excitation, respectively.

For an effective charge transfer in DSSC devices, in the excited state, the electronic charge has to move from the donor to the acceptor. The obtained charge density difference (CDD) and isosurfaces of *C*_+_ (green) and *C*_−_ (blue) functions (two centroids of charges associated with the positive and negative density regions) calculated^65,81^ with the Multiwfn code are presented in Fig. 6. The computed charge density difference (Δ*ρ*) between the first excited state (S_1_) and ground state (S_0_) was mapped to illustrate the intramolecular charge transfer (ICT). As can be seen in Fig. 6, there is an obvious left-to-right charge transfer character; the decreasing electron density on the electron donor group and π-spacer moiety, and increasing electron density on the electron acceptor group and π-spacer moiety. From the graph of *C*_+_ (green) and *C*_−_ (blue) functions, it is evident that the direction of electron transfer is from the donor group (electron donor) to the acceptor (electron acceptor).

### 2.8. Photovoltaic properties

Herein, we aimed to explore the effects of the chemical modification of dyes on the photovoltaic properties of the studied dyes. According to eqn (1), the *J*_SC_ and *V*_OC_ values must increase enough to improve the efficiency of the PCE. However, the amount of *J*_SC_ strongly depends on the dye regeneration (Δ*G*^reg^), the thermodynamic driving force (Δ*G*^inject^), and the light-harvesting efficiency (LHE) of dyes within the DSSC device.^54^ The efficiencies of the different dyes were determined through photovoltaic parameters, *viz*, LHE, Δ*G*^reg^, Δ*G*^inject^, *λ*_total,_ and *V*_OC_. The calculated values are summarized in Table 7.

**Table 7:** The calculated driving force of dye regeneration (Δ*G*^reg^, eV), the driving force of electron injection (Δ*G*^inject^, eV), open-circuit voltage (*eV*_OC_, eV), holes (*λ*_h_, eV), electrons (*λ*_e_, eV), (*λ*_total_, eV) total reorganization energies, LHE and excited-state lifetimes (*τ*, ns) of the dyes

Dye	Δ*G*^reg^	Δ *G*^inject^	*eV*_OC_	*λ*_h_	*λ*_e_	*λ*_total_	LHE	*τ*
JK-201	0.26	−1.63	1.05	0.146	0.360	0.506	0.992	1.53
JK-POZ	0.13	−1.70	1.03	0.243	0.270	0.513	0.990	1.68
JK-POZ-1	0.09	−1.68	0.90	0.235	0.254	0.489	0.993	1.63
JK-POZ-2	0.11	−1.63	0.70	0.235	0.255	0.490	0.990	1.81
JK-POZ-3	0.14	−1.62	0.57	0.233	0.229	0.462	0.988	1.88
JK-PTZ	0.24	−1.57	1.01	0.282	0.270	0.552	0.993	1.66
JK-PTZ-1	0.19	−1.57	0.88	0.261	0.254	0.515	0.990	1.61
JK-PTZ-2	0.21	−1.51	0.68	0.263	0.254	0.517	0.988	1.80
JK-PTZ-3	0.24	−1.50	0.55	0.263	0.228	0.491	0.980	1.89

**Fig. 7:**
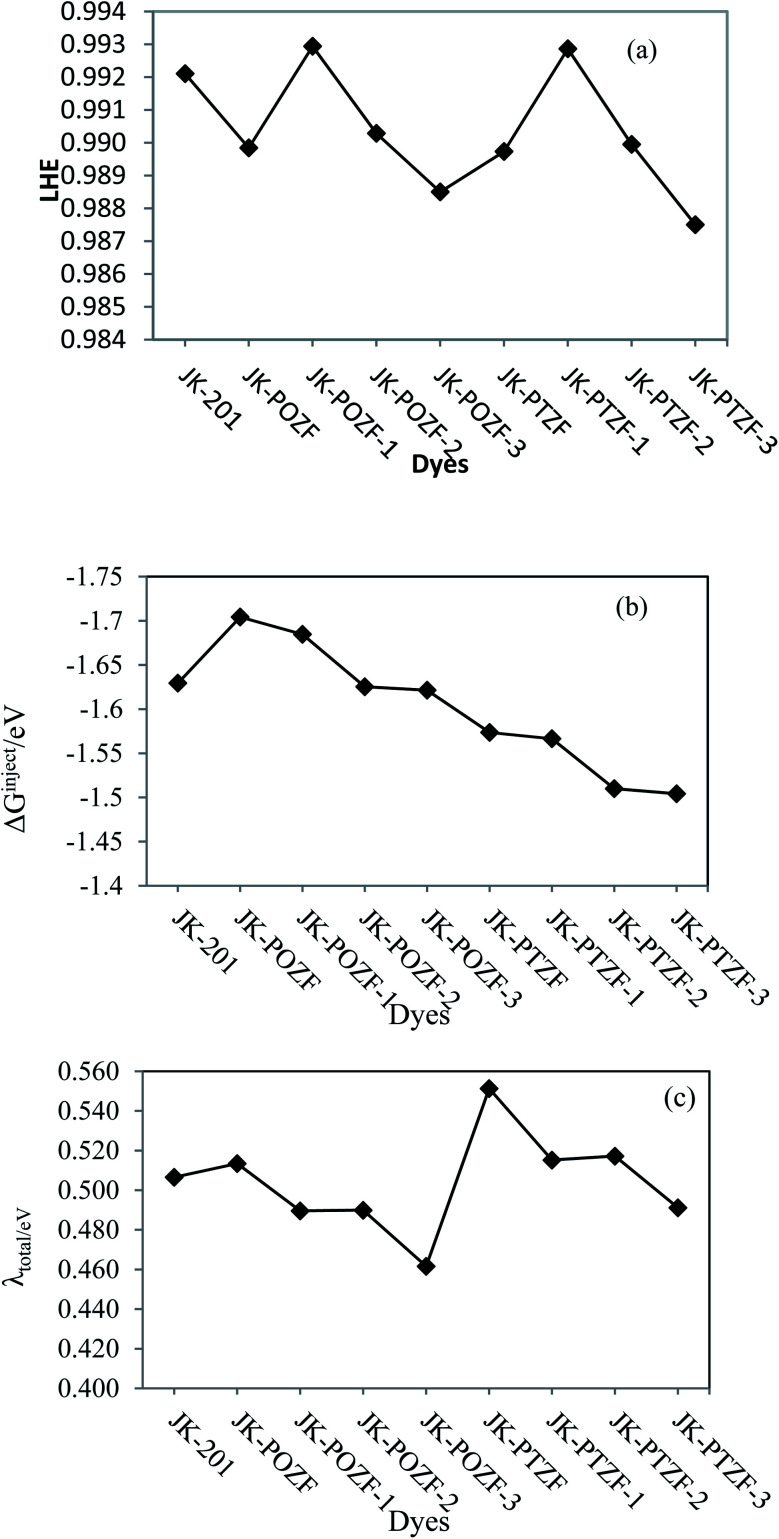
(a) The light-harvesting efficiency (LHE), (b) electronic injection-free energy (Δ*G*^inject^), and (c) reorganization energy (*λ*_total_) of the reference and designed dyes.

The LHE value illustrates the absorptivity of the dye molecule and is one of the main factors that determine the efficiency of the DSSCs device. The higher value of LHE and other parameters indicates an increase of the photocurrent response in sensitizer dye. From eqn (3), the sensitizer dyes with larger oscillator strength (*f*) values showed higher light-harvesting efficiencies in comparison to those with lower oscillator strength.^89^ The larger the oscillator strength of the dye at *λ*_max_, the higher the LHE. The calculated values of LHE given in Table 7 are great and in the range of 0.988–0.993; this means that all the dyes will give a similar photocurrent (Fig. 7a).

The Gibbs energy change for the electron injection of dyes (Δ*G*^inject^) is also the main parameter that affects the performance of the dye molecules. It determines the electron injection efficiency from the photoexcited dye sensitizer to the CB of the semiconductor substrate and, therefore, the efficiency of the DSSC system. When the amount of Δ*G*^inject^ for dyes is greater than 0.2 eV (Δ*G*^inject^ > 0.2), the injection efficiency of the electrons in the excited state is high and sufficient driving force is provided for the electron injection process.^90,91^ The calculated values of Δ*G*^inject^ for the investigated dyes are listed in Table 7, showing that the Δ*G*^inject^ values are negative and in the range of (−1.51 eV) to (−1.70 eV). This indicates that the injection of the electron from all the designed dyes to the TiO_2_ semiconductor is thermodynamically favorable, which leads to a high electron injection efficiency. The Δ*G*^inject^ of the reference and designed dyes follow the order of JK-POZ > JK-201 > JK-POZ-1 > JK-POZ-2 > JK-POZ-3 > JK-PTZ = JK-PTZ-1 > JK-PTZ-2 > JK-PTZ-3. Thus, it is predicted that the JK-POZ and JK-POZ(1–3) dyes would display maximum electron injection efficiency while JK-PTZ-2,3 dyes would exhibit the minimum electron injection efficiency in comparison with the other designed dyes under study. The results are graphically shown in Fig. 7b.

The dye molecules with high efficiency should possess a good charge transfer rate.^1^ Based on the Marcus theory,^92^ the standard Marcus/Hush model^93^ yields the following equation for the evaluation of the charge transfer rate:19
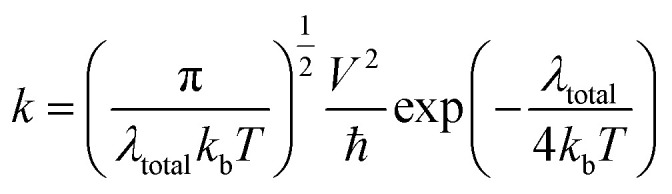
where *V* is the charge transfer coupling constant, *T* is the temperature, *λ*_total_ is the reorganization energy and *k*_b_ is the Boltzmann constant. In eqn (19), except for the reorganization energy, *λ*_total_, all the parameters on the right-hand side are constant. Thus, the electron transfer rate constant (*k*) depends only on *λ*_total_, which is the sum of the hole reorganization (*λ*_h_) and electron reorganization (*λ*_e_) energies, whose values can be estimated from (6) and (7), respectively. It can be seen from eqn (19) that an inverse relationship exists between the reorganization energy and the charge transfer-ability of the molecule. The electron, hole and total reorganization energies calculated according to eqn (6)–(8) for all dyes are summarized in Table 7. For an efficient system, the reorganization energy should be as small as possible to assure better charge transport and better electronic injection into the semiconductor conduction band. It can be seen from Table 7 that the total reorganization energies are in the following order: JK-POZ-3 < JK-POZ-1 < JK-POZ-2 < JK-PTZ-3 < JK-POZ < JK-201 < JK-PTZ-2 < JK-PTZ-1 < JK-PTZ. The substituted sensitizers JK-POZ-(1–3) and, JK-PTZ-(1–3) had the smallest values for total reorganization energy in comparison to the unsubstituted dye molecules, showing that they allowed much easier charge transport across the semiconductor conduction band interface and therefore can improve the efficiency of DSSCs. It needs to be mentioned that the dye JK-POZ-3 has the lowest reorganization energies among the reference and designed dyes, meaning that the JK-POZ-3 dye would show the best charge transfer-ability among the studied dyes. The results are graphically shown in Fig. 7c.

**Fig. 8:**
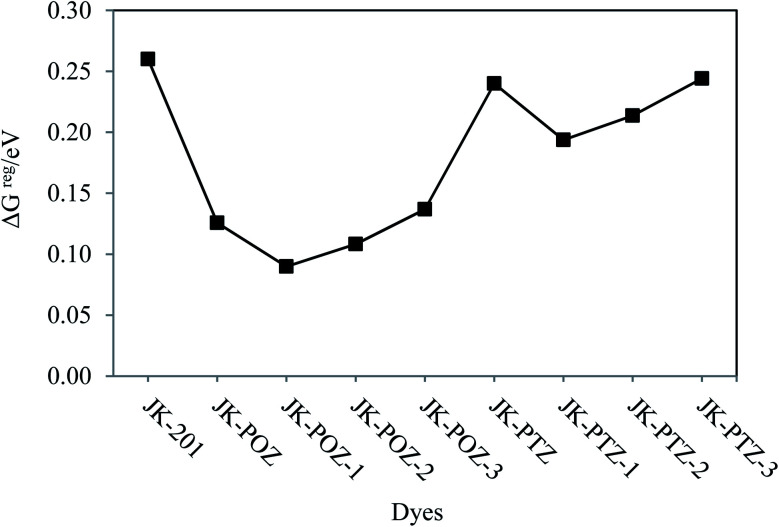
Variations of the driving force of regeneration (Δ*G*^reg^) of the reference and designed dyes.

Along with the mentioned parameters, the regeneration driving force (Δ*G*^reg^) also influences the efficiency of the DSSCs. To obtain faster regeneration of the sensitizers, low Δ*G*^reg^ is essential. The ΔG^reg^ can be estimated for the dye molecules using eqn (9). The calculated Δ*G*^reg^ values of all dyes are listed in Table 7 and are shown in Fig. 8. It can be seen that the free energies (in eV) of dye regeneration for all the sensitizers under consideration follow the trend JK-201(0.26) < JK-PTZ-3 = JK-PTZ (0.24) < JK-PTZ-2 (0.21) < JK-PTZ-1 (0.19) < JK-POZ-3 (0.14) < JK-POZ (0.13) < JK-POZ-2 (0.11) < JK-POZ-1 (0.09). This shows that all dyes have better chances of regeneration in comparison to the reference dye molecule (JK-201).

**Fig. 9:**
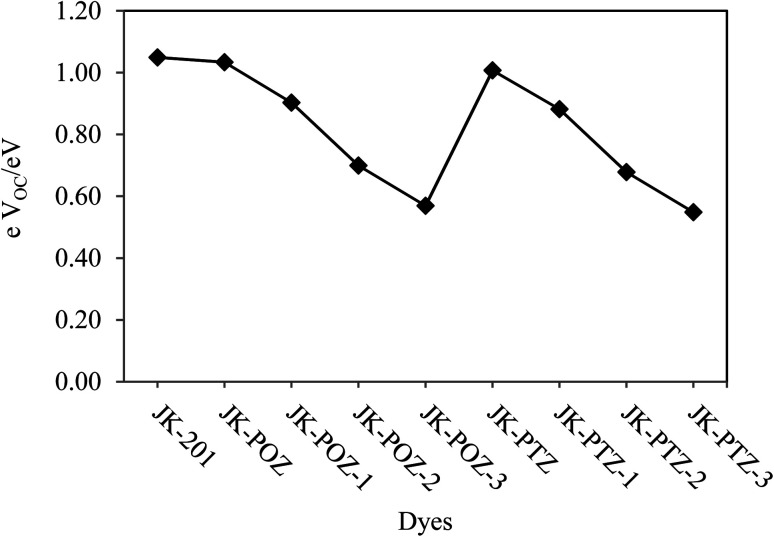
The *eV*_OC_ parameters of the reference and designed dyes.

The open-circuit voltage (electron-driving force) (*eV*_OC_) is an important parameter in DSSCs and is equal to the difference between the quasi-Fermi level of the electron in the TiO_2_ conduction band and the redox potential of the electrolyte. The Fermi level is defined by the potential of the conduction band (*E*_CB_) and the electron density in the TiO_2_.^94^ However, based on the electron injection from the LUMO level of the dye to the conduction band of TiO_2_, the *eV*_OC_ of the DSSCs system can be estimated by using eqn (10). The calculated *V*_OC_ values of the investigated dyes are listed in Table 7. The order of the values is as follows: JK-PTZ-3 < JK-POZ-3 < JK-PTZ-2 < JK-PTZ-1 < JK-POZ-1 < JK-PTZ < JK-POZ < JK-201. As can be seen, *eV*_OC_ decreased with the incorporation of the electron-withdrawing substituents. It was predicted that the JK-PTZ, JK-POZ and JK-201 dyes would show greater values of *eV*_OC_ in comparison to the other dyes. In addition, the JK-POZ-based substituted dyes have greater *eV*_OC_ than the JK-PTZ-based substituted ones. The results are graphically shown in Fig. 9.

### 2.9. Excited-state lifetime (*τ*)

The excited-state lifetime (*τ*) of the dye is one of the critical parameters for measuring the efficiency of charge transfer *via* the injection of the electron to the TiO_2_ in the excited state.^95^ It provides an estimation of the time for electron injection from the dye to the semiconductor substrate. The longer the excited state lifetime, the higher the optical stability of the dye in the excited state and the lower the light-emitting efficiency of the dye. After the electron injection, the dye is in a cationic form until regeneration by a redox mediator. The longer lifetime (*τ*) in the excited state indicates that the dye spends more time in the cationic form, which is more favorable for the charge transfer.^12,46^ The *τ* value is obtained by using the following equation:

20 *τ* = 1.499/*fE*^2^

where *E* is the excitation energy corresponding to the different excited states (cm^−1^) and *f* stands for the oscillator strength of the corresponding excited state.^96^

**Fig. 10:**
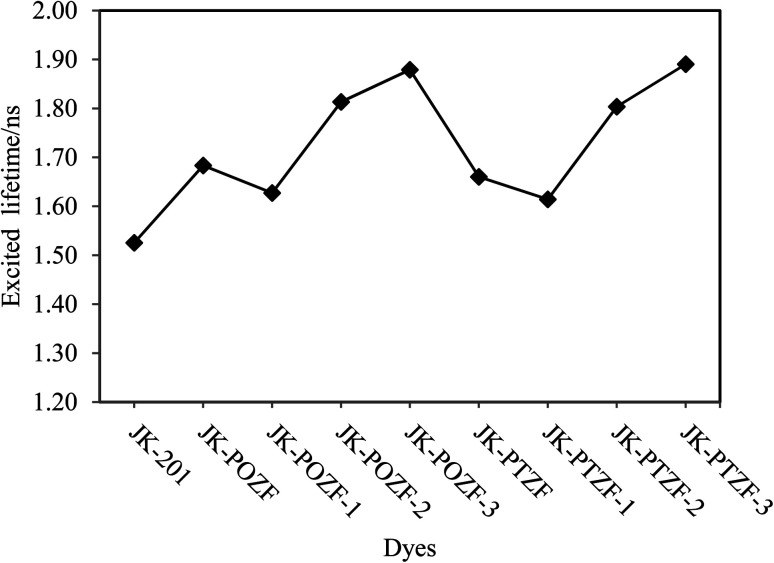
The calculated first excited-state lifetimes of the reference and designed dyes.

The calculated excited-state lifetimes of the investigated dyes are listed in Table 7 and are presented in Fig. 10. From Table 7, it can be seen that the excited-state lifetimes of the reference and designed dyes are in the following order: JK-PTZ-3 > JK-POZ-3 > JK-POZ-2 > JK-PTZ-2 > JK-POZ > JK-PTZ > JK-POZ-1 > JK-PTZ-1 > JK-201. The results show that the lifetimes of the designed dyes in the excited states are 0.365, 0.353, 0.288, 0.278, 0.158, 0.135, 0.102 and 0.089 ns, respectively, which are greater than that of the JK-201 dye. However, an increase in the excited state lifetime of the designed dyes will retard the charge recombination process and enhance the efficiency of the DSSCs. Besides, the JK-PTZ-3 and JK-POZ-3 dyes showed the longest excited-state lifetimes among the other dyes, indicating that these dyes could be used as candidates for high efficiency due to their excellent optical properties among the investigated dyes.

## 3. Conclusions

We have designed a series of D–π–A–A dyes by modification of the JK-201 reference dye by adding auxiliary heterocyclic acceptors with various functional groups and donor moieties that typically improved the photophysical properties of the designed dyes. DFT and TD-DFT methods were utilized to study the geometries and properties of the reference and designed dyes for DSSC devices. The calculated LUMO energy level gradually decreased with the incorporation of the electron-withdrawing substituents. All the electron-accepting substituted dyes showed a very narrow energy gap, leading to a broad UV-Vis absorption spectrum and red-shifts in wavelength when compared with the JK-201 dye. Among the dyes, JK-POZ-3 and JK-PTZ-3 dyes had significantly lower band gaps. The charge separation led to the increase in the *q*^CT^ value in the designed dyes relative to the reference dye. The light-harvesting efficiency (LHE), the driving force of injected electrons (Δ*G*^inject^), the lifetime of the excited state, *τ*, regeneration driving force (Δ*G*^reg^) and *eV*_OC_ values that most influence DSSC device performance were calculated for all dyes. As can be seen, *eV*_OC_ decreased with the incorporation of the electron-withdrawing substituents. Importantly, Δ*G*^reg^, *λ*_max_, *λ*_total_ and *τ* of JK-POZ-3, JK-PTZ-3 and JK-POZ-2 dyes, which are related to the vital photoelectrochemical properties, chemical reactivity and charge transfer, are superior to those of JK-201. All the designed dyes have better chances of regeneration in comparison to the reference dye molecule (JK-201). These organic dyes are promising dye candidates for extending the absorption wavelength and improving the overall energy conversion performances of DSSCs devices.

## Conflicts of interest

There are no conflicts of interest to declare.

## Supplementary Material

RA-012-D2RA00906D-s001
