# Supplementary material for: The role of the donor group and electron-accepting substitutions inserted in π-linkers in tuning the optoelectronic properties of D–π–A dye-sensitized solar cells: a DFT/TDDFT study
Source: RSC Adv. 2022 Apr 13;12(18):11557–73. doi: 10.1039/d2ra00906d (PMC9006569; doi:10.1039/d2ra00906d)

Electronic Supplementary Material (ESI) for RSC Advances.
This journal is © The Royal Society of Chemistry 2022

# Supplementary data file

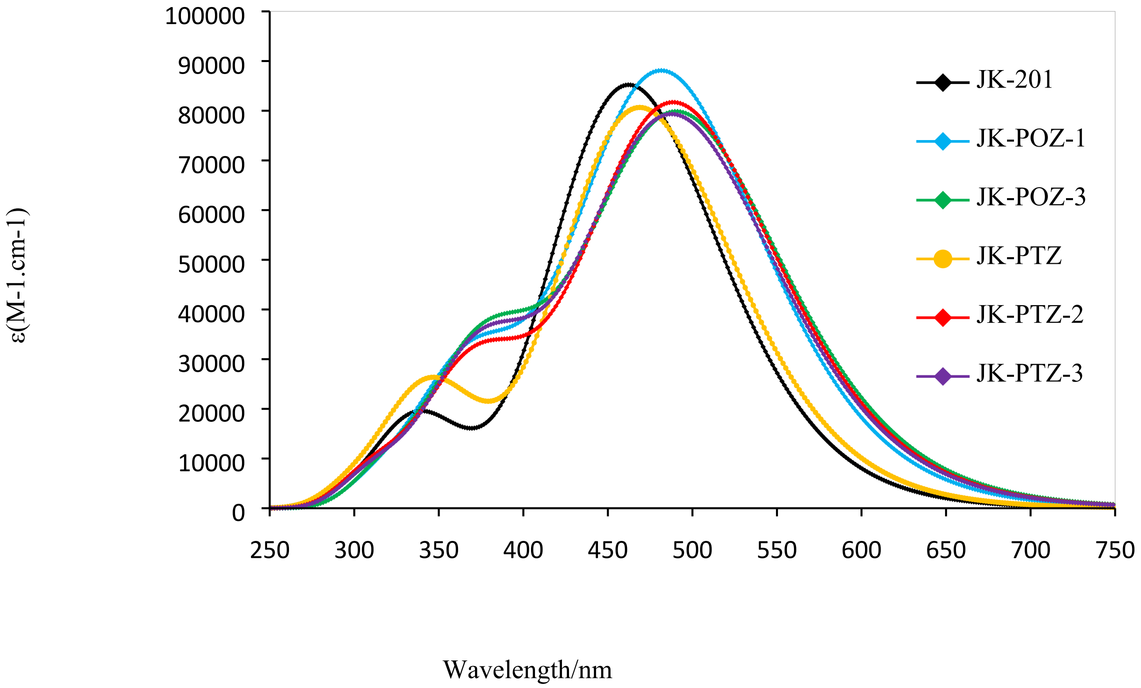


**Fig. S1.** Calculated absorption spectra of the reference and JK-POZ-1, JK-POZ-3 , JK-PTZ, JK-PTZ-2 and JK-PTZ-3 designed dyes at PCM/LC-wPBE/6-31++G(d,p) level of theory in THF solvent.

# The coordinate files for the molecular structures

| center Number | Atomic Number | Coordinates (Angsroms) X | Y | Z |
|---|---|---|---|---|
| 1 | 6 | 1.886998 | -1.24345 | -0.7451[illegible] |
| 2 | 6 | 2.498433 | -2.30476 | -1.3895[illegible] |
| 3 | 6 | 3.921289 | -2.28118 | -1.3361 |
| 4 | 6 | 4.444023 | -1.19158 | -0.6572[illegible] |
| 5 | 6 | 2.64249 | -4.03335 | -2.9591 |
| 6 | 6 | 3.959426 | -4.41608 | -2.3072 |
| 7 | 1 | 2.072486 | -4.92162 | -3.2396[illegible] |
| 8 | 1 | 4.602149 | -4.95969 | -3.0029 |
| 9 | 6 | 5.824092 | -0.86697 | -0.4265 |
| 10 | 6 | 6.943721 | -1.54576 | -0.8961[illegible] |
| 11 | 6 | 7.964229 | 0.205546 | 0.33357 |
| 12 | 6 | 8.141793 | -0.93856 | -0.4644[illegible] |
| 13 | 1 | 6.872824 | -2.42667 | -1.5174[illegible] |
| 14 | 6 | 9.150369 | 0.814997 | 0.76225 |
| 15 | 6 | 10.28133 | 0.139089 | 0.29402 |
| 16 | 1 | 9.205107 | 1.701138 | 1.38069 |
| 17 | 6 | 11.66808 | 0.393641 | 0.47664 |
| 18 | 6 | 12.30066 | 1.393953 | 1.17848 |
| 19 | 6 | 13.78173 | 1.394614 | 1.18409 |
| 20 | 6 | 11.6031 | 2.412182 | 1.88837 |
| 21 | 8 | 14.48211 | 0.575913 | 0.61393 |
| 22 | 8 | 14.29804 | 2.419686 | 1.90289 |
| 23 | 1 | 15.26502 | 2.341238 | 1.85143 |
| 24 | 1 | 12.35742 | -0.29769 | -0.0046[illegible] |
| 25 | 16 | 9.826426 | -1.27327 | -0.6933 |
| 26 | 16 | 6.264925 | 0.539658 | 0.56257 |
| 27 | 16 | 3.122663 | -0.19863 | -0.0585 |
| 28 | 7 | 10.996 | 3.228869 | 2.45756 |
| 29 | 8 | 1.819179 | -3.30802 | -2.0335[illegible] |
| 30 | 8 | 4.69453 | -3.23824 | -1.9278 |
| 31 | 1 | 2.819558 | -3.4182 | -3.8511[illegible] |
| 32 | 1 | 3.782323 | -5.03206 | -1.4166 |
| 33 | 6 | 0.469892 | -0.9309 | -0.5896[illegible] |
| 34 | 6 | 0.043183 | 0.353901 | -0.1908[illegible] |
| 35 | 6 | -0.53248 | -1.89884 | -0.8188[illegible] |
| 36 | 6 | -1.30054 | 0.65745 | -0.0197[illegible] |
| 37 | 1 | 0.774457 | 1.136023 | -0.0077[illegible] |
| 38 | 6 | -1.87967 | -1.59524 | -0.6624[illegible] |
| 39 | 1 | -0.25203 | -2.89694 | -1.1304[illegible] |
| 40 | 6 | -2.29346 | -0.31286 | -0.2562 |
| 41 | 1 | -1.58807 | 1.654366 | 0.29578 |
| 42 | 1 | -2.62332 | -2.36121 | -0.8541[illegible] |
| 43 | 7 | -3.66173 | -0.00713 | -0.0898 |
| 44 | 6 | -4.56917 | -0.99403 | 0.3941 |
| 45 | 6 | -4.23363 | -1.77967 | 1.51201 |
| 46 | 6 | -5.8122 | -1.17923 | -0.2401[illegible] |
| 47 | 6 | -5.11263 | -2.75075 | 1.99186 |
| 48 | 1 | -3.27753 | -1.62293 | 2.00078 |
| 49 | 6 | -6.6923 | -2.13619 | 0.24840 |
| 50 | 1 | -6.06352 | -0.56963 | -1.1031[illegible] |
| 51 | 6 | -6.34778 | -2.9297 | 1.36162 |
| 52 | 1 | -4.83461 | -3.34627 | 2.8570[illegible] |
| 53 | 6 | -8.07907 | -2.50922 | -0.2787[illegible] |
| 54 | 6 | -7.45469 | -3.85195 | 1.63971 |
| 55 | 6 | -8.47427 | -3.62303 | 0.69219 |
| 56 | 6 | -7.60688 | -4.83109 | 2.62618 |
| 57 | 6 | -9.64626 | -4.37234 | 0.72909 |
| 58 | 6 | -8.78734 | -5.57974 | 2.65862 |
| 59 | 1 | -6.82443 | -5.01193 | 3.35816 |
| 60 | 6 | -9.80027 | -5.35362 | 1.71838 |
| 61 | 1 | -10.4393 | -4.2057 | 0.00436 |
| 62 | 1 | -8.92018 | -6.34346 | 3.41955 |
| 63 | 1 | -10.712 | -5.94283 | 1.75606 |
| 64 | 6 | -4.15516 | 1.292607 | -0.4059[illegible] |
| 65 | 6 | -3.79168 | 1.915297 | -1.6139[illegible] |
| 66 | 6 | -5.01783 | 1.954596 | 0.48792 |
| 67 | 6 | -4.26603 | 3.188174 | -1.9317[illegible] |
| 68 | 1 | -3.13341 | 1.393235 | -2.3008[illegible] |
| 69 | 6 | -5.50119 | 3.21538 | 0.16279 |
| 70 | 1 | -5.29168 | 1.468022 | 1.41948 |
| 71 | 6 | -5.12635 | 3.840942 | -1.0438[illegible] |
| 72 | 1 | -3.97345 | 3.651737 | -2.8698[illegible] |
| 73 | 6 | -6.43559 | 4.113529 | 0.97526 |
| 74 | 6 | -5.77442 | 5.155797 | -1.1104 |
| 75 | 6 | -6.54241 | 5.33376 | 0.05927 |
| 76 | 6 | -5.72737 | 6.152864 | -2.0894[illegible] |
| 77 | 6 | -7.26355 | 6.508162 | 0.25205 |
| 78 | 6 | -6.45527 | 7.329913 | -1.8904[illegible] |
| 79 | 1 | -5.13621 | 6.02146 | -2.9916[illegible] |
| 80 | 6 | -7.21801 | 7.507972 | -0.7295 |

| center Number | Atomic Number | Coordinates (Angsroms) X | Y | Z |
|---|---|---|---|---|
| 81 | 1 | -7.85842 | 6.657864 | 1.149624 |
| 82 | 1 | -6.42853 | 8.113517 | -2.6422 |
| 83 | 1 | -7.77801 | 8.427883 | -0.58843 |
| 84 | 6 | -5.81397 | 4.485751 | 2.341403 |
| 85 | 6 | -7.81326 | 3.445102 | 1.189572 |
| 86 | 6 | -9.05451 | -1.31265 | -0.19308 |
| 87 | 6 | -8.00943 | -3.0271 | -1.73413 |
| 88 | 1 | -7.32337 | -3.87535 | -1.81744 |
| 89 | 1 | -8.99852 | -3.35179 | -2.07472 |
| 90 | 1 | -7.66323 | -2.23698 | -2.40925 |
| 91 | 1 | -8.72159 | -0.49547 | -0.842 |
| 92 | 1 | -10.0583 | -1.61101 | -0.51415 |
| 93 | 1 | -9.12217 | -0.93267 | 0.830697 |
| 94 | 1 | -7.71175 | 2.534628 | 1.789976 |
| 95 | 1 | -8.49188 | 4.123557 | 1.717683 |
| 96 | 1 | -8.27362 | 3.177016 | 0.233872 |
| 97 | 1 | -4.83681 | 4.960805 | 2.212885 |
| 98 | 1 | -6.46416 | 5.18103 | 2.883167 |
| 99 | 1 | -5.6834 | 3.593029 | 2.962604 |

## JK-201

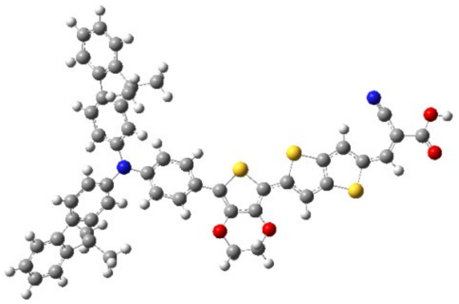

| center | Atomic | Coordinates (Angsroms) | | |
|---|---|---|---|---|

| center Number | Atomic Number | Coordinates (Angsroms) X | Y | Z |
|---|---|---|---|---|
| 1 | 6 | -0.36914 | -1.44105 | -0.32867 |
| 2 | 6 | -1.48093 | -2.23986 | -0.45742 |
| 3 | 6 | -2.62692 | -1.39512 | -0.32357 |
| 4 | 1 | -1.47837 | -3.31102 | -0.58933 |
| 5 | 6 | -2.1412 | -0.10863 | -0.13844 |
| 6 | 6 | 1.037866 | -1.69736 | -0.35012 |
| 7 | 6 | 1.679881 | -2.90575 | -0.55604 |
| 8 | 6 | 3.095235 | -2.8157 | -0.5078 |
| 9 | 6 | 3.572525 | -1.53138 | -0.28014 |
| 10 | 6 | 1.875283 | -5.10775 | -1.30321 |
| 11 | 6 | 3.192963 | -5.15286 | -0.54708 |
| 12 | 1 | 1.330711 | -6.04787 | -1.19288 |
| 13 | 1 | 3.854439 | -5.91871 | -0.95723 |
| 14 | 6 | 4.937512 | -1.1006 | -0.17608 |
| 15 | 6 | 6.083366 | -1.86143 | -0.38746 |
| 16 | 6 | 7.03517 | 0.237925 | 0.166792 |
| 17 | 6 | 7.25678 | -1.10383 | -0.19208 |
| 18 | 1 | 6.046814 | -2.90326 | -0.67135 |
| 19 | 6 | 8.196723 | 0.99676 | 0.35718 |
| 20 | 6 | 9.353499 | 0.240019 | 0.144937 |
| 21 | 1 | 8.217147 | 2.042373 | 0.634619 |
| 22 | 6 | 10.72919 | 0.588589 | 0.224911 |
| 23 | 6 | 11.32241 | 1.789494 | 0.54111 |
| 24 | 6 | 12.80234 | 1.842452 | 0.540989 |
| 25 | 6 | 10.58548 | 2.964311 | 0.86342 |
| 26 | 8 | 13.53437 | 0.902742 | 0.281956 |
| 27 | 8 | 13.27855 | 3.068223 | 0.865101 |
| 28 | 1 | 14.24796 | 3.009587 | 0.840041 |
| 29 | 1 | 11.44517 | -0.20154 | 0.006183 |
| 30 | 16 | 8.953535 | -1.43959 | -0.29777 |
| 31 | 16 | 5.323896 | 0.572822 | 0.27424 |
| 32 | 16 | 2.21443 | -0.42076 | -0.11538 |
| 33 | 7 | 9.946574 | 3.904828 | 1.121952 |
| 34 | 8 | 1.019288 | -4.08553 | -0.76677 |
| 35 | 8 | 3.899591 | -3.90539 | -0.68222 |
| 36 | 1 | 2.051406 | -4.91159 | -2.36884 |
| 37 | 1 | 3.018709 | -5.35311 | 0.517534 |
| 38 | 8 | -0.76581 | -0.14449 | -0.13249 |
| 39 | 6 | -4.02399 | -1.87656 | -0.334 |
| 40 | 6 | -4.92631 | -1.52067 | 0.687412 |
| 41 | 6 | -4.47003 | -2.74619 | -1.33897 |
| 42 | 6 | -6.21958 | -2.01757 | 0.691162 |
| 43 | 1 | -4.6226 | -0.86443 | 1.495859 |
| 44 | 6 | -5.7779 | -3.23891 | -1.33588 |
| 45 | 1 | -3.79691 | -3.02921 | -2.14226 |
| 46 | 6 | -6.66901 | -2.88529 | -0.32048 |
| 47 | 1 | -6.10876 | -3.90758 | -2.12661 |
| 48 | 6 | -8.4063 | -1.88384 | 1.597127 |
| 49 | 6 | -8.89703 | -2.74381 | 0.600128 |
| 50 | 1 | -8.3446 | -3.8334 | -1.06812 |
| 51 | 6 | -9.26842 | -1.24936 | 2.480644 |
| 52 | 6 | -10.2755 | -2.9525 | 0.508825 |
| 53 | 1 | -8.8483 | -0.59034 | 3.233339 |
| 54 | 6 | -10.6481 | -1.47295 | 2.389726 |
| 55 | 6 | -11.1481 | -2.32346 | 1.403607 |
| 56 | 1 | -10.6627 | -3.61739 | -0.2594 |
| 57 | 1 | -11.3198 | -0.98121 | 3.085742 |
| 58 | 1 | -12.2157 | -2.50189 | 1.322376 |
| 59 | 6 | -2.73517 | 1.213919 | 0.00671 |
| 60 | 6 | -2.01082 | 2.25919 | 0.60654 |
| 61 | 6 | -4.03126 | 1.491678 | -0.47985 |
| 62 | 6 | -2.56509 | 3.532114 | 0.73141 |
| 63 | 1 | -1.01009 | 2.074715 | 0.979393 |
| 64 | 6 | -4.57697 | 2.754887 | -0.34065 |
| 65 | 1 | -4.62042 | 0.731044 | -0.97713 |
| 66 | 6 | -3.85753 | 3.798814 | 0.270293 |
| 67 | 1 | -1.9939 | 4.327024 | 1.204044 |
| 68 | 6 | -6.32124 | 4.257493 | -0.92472 |
| 69 | 6 | -5.6345 | 5.325444 | -0.32228 |
| 70 | 1 | -3.89173 | 5.82385 | 0.680239 |
| 71 | 6 | -7.49247 | 4.474873 | -1.63719 |
| 72 | 6 | -6.14855 | 6.618027 | -0.4534 |
| 73 | 6 | -8.0066 | 5.772383 | -1.75573 |
| 74 | 1 | -7.99176 | 3.623065 | -2.08725 |
| 75 | 6 | -7.33297 | 6.840825 | -1.16393 |
| 76 | 1 | -5.62253 | 7.448538 | 0.010873 |
| 77 | 1 | -8.92569 | 5.938414 | -2.30812 |
| 78 | 1 | -7.72101 | 7.85074 | -1.25115 |
| 79 | 8 | -7.04404 | -1.67103 | 1.748739 |
| 80 | 8 | -5.86824 | 2.953061 | -0.80215 |
| 81 | 7 | -7.97609 | -3.37709 | -0.246 |
| 82 | 7 | -4.47109 | 5.044028 | 0.405191 |

# JK-POZ

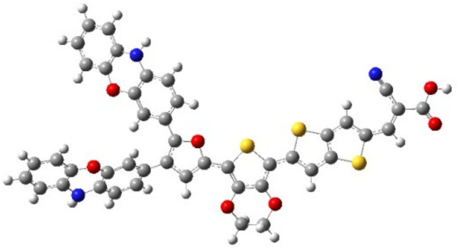

| center Number | Atomic Number | Coordinates (Angsroms) X | Y | Z |
|---|---|---|---|---|
| 1 | 6 | -2.11411 | -1.66067 | -0.30932 |
| 2 | 6 | -3.30142 | -2.35044 | -0.37755 |
| 3 | 6 | -4.3557 | -1.39054 | -0.26256 |
| 4 | 1 | -3.40708 | -3.42152 | -0.4574 |
| 5 | 6 | -3.74212 | -0.15172 | -0.14973 |
| 6 | 6 | -0.73949 | -2.05605 | -0.34686 |
| 7 | 6 | -0.22354 | -3.32934 | -0.5066 |
| 8 | 6 | 1.195372 | -3.37743 | -0.49138 |
| 9 | 6 | 1.801109 | -2.13856 | -0.3359 |
| 10 | 6 | -0.2623 | -5.5737 | -1.14552 |
| 11 | 6 | 1.063501 | -5.71158 | -0.41505 |
| 12 | 1 | -0.89304 | -6.44904 | -0.97642 |
| 13 | 1 | 1.635534 | -6.55926 | -0.79801 |
| 14 | 6 | 3.205328 | -1.84045 | -0.28135 |
| 15 | 6 | 4.266791 | -2.71357 | -0.49292 |
| 16 | 6 | 5.431823 | -0.70206 | -0.03871 |
| 17 | 6 | 5.512093 | -2.06399 | -0.35281 |
| 18 | 1 | 4.123502 | -3.75605 | -0.73849 |
| 19 | 6 | 6.676958 | -0.05869 | 0.092833 |
| 20 | 6 | 7.738975 | -0.92825 | -0.12036 |
| 21 | 1 | 6.808177 | 0.990567 | 0.331054 |
| 22 | 16 | 7.172768 | -2.57051 | -0.49007 |
| 23 | 16 | 3.766856 | -0.19929 | 0.09977 |
| 24 | 16 | 0.562972 | -0.89259 | -0.19971 |
| 25 | 8 | -1.00085 | -4.4473 | -0.64497 |
| 26 | 8 | 1.885197 | -4.54922 | -0.62983 |
| 27 | 1 | -0.09449 | -5.4483 | -2.22315 |
| 28 | 1 | 0.897066 | -5.84047 | 0.661899 |
| 29 | 8 | -2.37673 | -0.32347 | -0.16869 |
| 30 | 6 | 9.144071 | -0.63931 | -0.07153 |
| 31 | 6 | 10.20464 | -1.51405 | -0.29571 |
| 32 | 16 | 9.71346 | 0.976061 | 0.306241 |
| 33 | 6 | 11.43613 | -0.8699 | -0.16014 |
| 34 | 1 | 10.10252 | -2.56235 | -0.54512 |
| 35 | 6 | 11.40306 | 0.485927 | 0.163738 |
| 36 | 6 | 12.55309 | 1.293358 | 0.339111 |
| 37 | 6 | 12.67213 | 2.625109 | 0.655003 |
| 38 | 6 | 11.55228 | 3.478335 | 0.875439 |
| 39 | 6 | 14.04058 | 3.183325 | 0.767542 |
| 40 | 7 | 10.62158 | 4.158164 | 1.051671 |
| 41 | 1 | 14.96942 | 4.778152 | 1.13434 |
| 42 | 8 | 15.0669 | 2.548511 | 0.600466 |
| 43 | 8 | 14.03996 | 4.500162 | 1.081975 |
| 44 | 1 | 13.4997 | 0.774795 | 0.201225 |
| 45 | 6 | -5.79309 | -1.73088 | -0.22128 |
| 46 | 6 | -6.34708 | -2.59978 | -1.17171 |
| 47 | 6 | -6.6307 | -1.23798 | 0.798312 |
| 48 | 6 | -7.69654 | -2.95952 | -1.11769 |
| 49 | 1 | -5.72495 | -2.98704 | -1.9726 |
| 50 | 6 | -7.96596 | -1.60343 | 0.852998 |
| 51 | 1 | -6.24412 | -0.5765 | 1.566067 |
| 52 | 6 | -8.52325 | -2.47038 | -0.10407 |
| 53 | 1 | -8.11086 | -3.6298 | -1.86664 |
| 54 | 6 | -10.1058 | -1.21062 | 1.795118 |
| 55 | 6 | -10.7031 | -2.0651 | 0.852992 |
| 56 | 1 | -10.3019 | -3.28233 | -0.76948 |
| 57 | 6 | -10.8794 | -0.45245 | 2.662959 |
| 58 | 6 | -12.0973 | -2.14106 | 0.800652 |
| 59 | 6 | -12.2762 | -0.54309 | 2.611736 |
| 60 | 1 | -10.3782 | 0.197511 | 3.37263 |
| 61 | 1 | -12.5667 | -2.80085 | 0.07506 |
| 62 | 6 | -12.8815 | -1.3867 | 1.680192 |
| 63 | 1 | -12.879 | 0.045467 | 3.295639 |
| 64 | 1 | -13.9631 | -1.46273 | 1.629902 |
| 65 | 6 | -4.20008 | 1.228626 | -0.05809 |
| 66 | 6 | -3.36532 | 2.223285 | 0.481147 |
| 67 | 6 | -5.47158 | 1.610974 | -0.53833 |
| 68 | 6 | -3.78948 | 3.549314 | 0.55378 |
| 69 | 1 | -2.38062 | 1.957617 | 0.847672 |
| 70 | 6 | -5.88781 | 2.927105 | -0.45103 |
| 71 | 1 | -6.14185 | 0.890458 | -0.99066 |
| 72 | 6 | -5.05796 | 3.921353 | 0.099423 |
| 73 | 1 | -3.13415 | 4.304518 | 0.980149 |
| 74 | 6 | -7.48433 | 4.567036 | -1.08471 |
| 75 | 6 | -6.68489 | 5.588215 | -0.54316 |
| 76 | 1 | -4.88458 | 5.956483 | 0.411656 |
| 77 | 6 | -8.63925 | 4.866613 | -1.79401 |
| 78 | 6 | -7.06952 | 6.918121 | -0.7321 |
| 79 | 6 | -9.02331 | 6.202013 | -1.97048 |
| 80 | 1 | -9.22809 | 4.048665 | -2.19608 |
| 81 | 1 | -6.45597 | 7.712916 | -0.31511 |

| center Number | Atomic Number | Coordinates (Angsroms) X | Y | Z |
|---|---|---|---|---|
| 82 | 6 | -8.23708 | 7.224479 | -1.43943 |
| 83 | 1 | -9.92999 | 6.432863 | -2.52007 |
| 84 | 1 | -8.52383 | 8.262927 | -1.57183 |
| 85 | 7 | -5.54402 | 5.226384 | 0.184549 |
| 86 | 7 | -9.87005 | -2.82621 | 0.021307 |
| 87 | 8 | -8.726 | -1.12583 | 1.907908 |
| 88 | 8 | -7.16205 | 3.231166 | -0.90253 |
| 89 | 9 | 12.59925 | -1.52307 | -0.3334 |

# JK-POZ-1

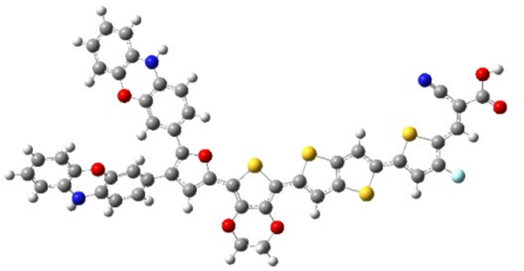

| center Number | Atomic Number | Coordinates (Angsroms) X | Y | Z |
|---|---|---|---|---|
| 1 | 6 | -2.72475 | -1.61886 | -0.29518 |
| 2 | 6 | -3.89758 | -2.33291 | -0.36633 |
| 3 | 6 | -4.97157 | -1.39449 | -0.25923 |
| 4 | 1 | -3.9811 | -3.40617 | -0.44316 |
| 5 | 6 | -4.38381 | -0.1429 | -0.14778 |
| 6 | 6 | -1.34251 | -1.98647 | -0.32491 |
| 7 | 6 | -0.80038 | -3.25003 | -0.47683 |
| 8 | 6 | 0.618887 | -3.26958 | -0.45468 |
| 9 | 6 | 1.199095 | -2.01788 | -0.30198 |
| 10 | 6 | -0.79112 | -5.49717 | -1.10695 |
| 11 | 6 | 0.53353 | -5.60568 | -0.36956 |
| 12 | 1 | -1.40509 | -6.38424 | -0.93736 |
| 13 | 1 | 1.124507 | -6.4429 | -0.74665 |
| 14 | 6 | 2.596466 | -1.69192 | -0.24262 |
| 15 | 6 | 3.676156 | -2.5468 | -0.43748 |
| 16 | 6 | 4.798974 | -0.50772 | -0.00024 |
| 17 | 6 | 4.907477 | -1.87169 | -0.29771 |
| 18 | 1 | 3.554613 | -3.59471 | -0.67127 |
| 19 | 6 | 6.030691 | 0.160621 | 0.131711 |
| 20 | 6 | 7.110534 | -0.69114 | -0.06433 |
| 21 | 1 | 6.140371 | 1.214837 | 0.358766 |
| 22 | 16 | 6.578175 | -2.34823 | -0.41842 |
| 23 | 16 | 3.123882 | -0.03552 | 0.121851 |
| 24 | 16 | -0.06447 | -0.79643 | -0.1763 |
| 25 | 8 | -1.55449 | -4.3837 | -0.61441 |
| 26 | 8 | 1.332718 | -4.42759 | -0.58452 |
| 27 | 1 | -0.62071 | -5.37257 | -2.18424 |
| 28 | 1 | 0.364557 | -5.73397 | 0.707038 |
| 29 | 8 | -3.01519 | -0.28682 | -0.16021 |
| 30 | 6 | 8.509681 | -0.37535 | -0.00987 |
| 31 | 6 | 9.590613 | -1.23215 | -0.21108 |
| 32 | 16 | 9.061396 | 1.243161 | 0.342039 |
| 33 | 6 | 10.83256 | -0.59414 | -0.08386 |
| 34 | 1 | 9.483925 | -2.28385 | -0.44247 |
| 35 | 6 | 10.7522 | 0.773659 | 0.219202 |
| 36 | 6 | 11.85615 | 1.654243 | 0.401665 |
| 37 | 6 | 11.89107 | 2.99416 | 0.697647 |
| 38 | 6 | 10.72976 | 3.797598 | 0.888304 |
| 39 | 6 | 13.22878 | 3.629742 | 0.823439 |
| 40 | 7 | 9.771828 | 4.443984 | 1.041957 |
| 41 | 1 | 14.05964 | 5.280448 | 1.1796 |
| 42 | 8 | 14.2882 | 3.047654 | 0.682799 |
| 43 | 8 | 13.14885 | 4.947826 | 1.116113 |
| 44 | 1 | 12.84 | 1.206102 | 0.291669 |
| 45 | 6 | -6.40191 | -1.76411 | -0.2235 |
| 46 | 6 | -6.9342 | -2.64436 | -1.17585 |
| 47 | 6 | -7.25336 | -1.2882 | 0.792688 |
| 48 | 6 | -8.27623 | -3.03158 | -1.12703 |
| 49 | 1 | -6.30112 | -3.019 | -1.97416 |
| 50 | 6 | -8.58107 | -1.68089 | 0.842285 |
| 51 | 1 | -6.88341 | -0.61874 | 1.561735 |
| 52 | 6 | -9.11674 | -2.55925 | -0.11678 |
| 53 | 1 | -8.67382 | -3.71033 | -1.87741 |
| 54 | 6 | -10.7322 | -1.33205 | 1.77614 |
| 55 | 6 | -11.3081 | -2.19873 | 0.831908 |
| 56 | 1 | -10.8757 | -3.40795 | -0.78867 |
| 57 | 6 | -11.5245 | -0.59007 | 2.641061 |
| 58 | 6 | -12.7002 | -2.30354 | 0.774367 |
| 59 | 6 | -12.9189 | -0.7096 | 2.584642 |
| 60 | 1 | -11.0395 | 0.070099 | 3.352571 |
| 61 | 1 | -13.1531 | -2.97292 | 0.04711 |
| 62 | 6 | -13.5031 | -1.56555 | 1.650921 |
| 63 | 1 | -13.5363 | -0.13367 | 3.266268 |
| 64 | 1 | -14.5827 | -1.66396 | 1.596628 |
| 65 | 6 | -4.87035 | 1.227917 | -0.06245 |
| 66 | 6 | -4.05703 | 2.241921 | 0.473601 |
| 67 | 6 | -6.14912 | 1.581809 | -0.54533 |
| 68 | 6 | -4.50883 | 3.55904 | 0.540677 |
| 69 | 1 | -3.06739 | 1.998338 | 0.842142 |
| 70 | 6 | -6.59281 | 2.889255 | -0.46359 |
| 71 | 1 | -6.80386 | 0.845742 | -0.99531 |
| 72 | 6 | -5.78439 | 3.902795 | 0.083721 |
| 73 | 1 | -3.86985 | 4.329402 | 0.964716 |
| 74 | 6 | -8.22381 | 4.492856 | -1.10296 |
| 75 | 6 | -7.44634 | 5.532582 | -0.56477 |
| 76 | 1 | -5.65422 | 5.942286 | 0.389081 |
| 77 | 6 | -9.3853 | 4.765419 | -1.8124 |
| 78 | 6 | -7.85964 | 6.853338 | -0.75722 |
| 79 | 6 | -9.79813 | 6.091721 | -1.99246 |
| 80 | 1 | -9.95659 | 3.933827 | -2.21179 |
| 81 | 1 | -7.26319 | 7.662361 | -0.34281 |
| 82 | 6 | -9.03387 | 7.132424 | -1.46479 |
| 83 | 1 | -10.7099 | 6.301389 | -2.54218 |
| 84 | 1 | -9.34302 | 8.164059 | -1.59998 |
| 85 | 7 | -6.29752 | 5.197564 | 0.163248 |
| 86 | 7 | -10.4564 | -2.94243 | 0.003374 |
| 87 | 8 | -9.35479 | -1.21867 | 1.894018 |
| 88 | 8 | -7.87262 | 3.164736 | -0.91745 |
| 89 | 6 | 12.13058 | -1.3342 | -0.26189 |
| 90 | 9 | 12.87869 | -0.82104 | -1.27183 |
| 91 | 9 | 12.90284 | -1.28993 | 0.853798 |
| 92 | 9 | 11.92232 | -2.64073 | -0.5477 |

## JK-POZ-2

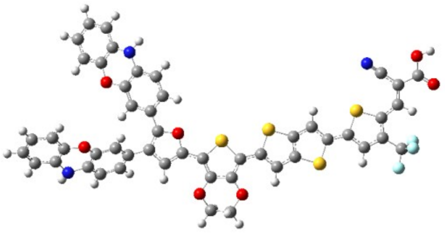

| center Number | Atomic Number | Coordinates (Angsroms) X | Y | Z |
|---|---|---|---|---|
| 1 | 6 | -2.22232 | -1.64976 | -0.30471 |
| 2 | 6 | -3.4056 | -2.34667 | -0.37416 |
| 3 | 6 | -4.46556 | -1.39319 | -0.26243 |
| 4 | 1 | -3.50478 | -3.41846 | -0.45288 |
| 5 | 6 | -3.85937 | -0.15035 | -0.15005 |
| 6 | 6 | -0.84583 | -2.03731 | -0.3392 |
| 7 | 6 | -0.32267 | -3.30833 | -0.49668 |
| 8 | 6 | 1.096061 | -3.34864 | -0.47903 |
| 9 | 6 | 1.694916 | -2.10577 | -0.3242 |
| 10 | 6 | -0.34818 | -5.55266 | -1.13566 |
| 11 | 6 | 0.97693 | -5.68379 | -0.40281 |
| 12 | 1 | -0.9745 | -6.4314 | -0.96798 |
| 13 | 1 | 1.554754 | -6.52755 | -0.7856 |
| 14 | 6 | 3.096787 | -1.80066 | -0.26807 |
| 15 | 6 | 4.163316 | -2.67085 | -0.46918 |
| 16 | 6 | 5.316985 | -0.64967 | -0.02876 |
| 17 | 6 | 5.404527 | -2.01424 | -0.33113 |
| 18 | 1 | 4.025653 | -3.71606 | -0.70605 |
| 19 | 6 | 6.558741 | -0.00017 | 0.101298 |
| 20 | 6 | 7.625109 | -0.86724 | -0.10114 |
| 21 | 1 | 6.684229 | 1.051689 | 0.331185 |
| 22 | 16 | 7.067581 | -2.515 | -0.45884 |
| 23 | 16 | 3.649426 | -0.15336 | 0.100109 |
| 24 | 16 | 0.449641 | -0.86639 | -0.19067 |
| 25 | 8 | -1.09364 | -4.43029 | -0.6358 |
| 26 | 8 | 1.792569 | -4.51616 | -0.61461 |
| 27 | 1 | -0.17936 | -5.42577 | -2.21292 |
| 28 | 1 | 0.80947 | -5.81456 | 0.673675 |
| 29 | 8 | -2.49308 | -0.3139 | -0.16624 |
| 30 | 6 | 9.028975 | -0.57177 | -0.05027 |
| 31 | 6 | 10.09625 | -1.43843 | -0.26157 |
| 32 | 16 | 9.600302 | 1.043354 | 0.31308 |
| 33 | 6 | 11.35179 | -0.81065 | -0.13322 |
| 34 | 1 | 9.982803 | -2.48835 | -0.50113 |
| 35 | 6 | 11.28176 | 0.559792 | 0.181198 |
| 36 | 6 | 12.4115 | 1.404203 | 0.360434 |
| 37 | 6 | 12.48638 | 2.740015 | 0.665208 |
| 38 | 6 | 11.34388 | 3.566921 | 0.868716 |
| 39 | 6 | 13.84055 | 3.341802 | 0.78608 |
| 40 | 7 | 10.3975 | 4.227818 | 1.031801 |
| 41 | 1 | 14.71259 | 4.970282 | 1.148123 |
| 42 | 8 | 14.88291 | 2.733694 | 0.633821 |
| 43 | 8 | 13.79392 | 4.659087 | 1.088305 |
| 44 | 1 | 13.37716 | 0.916573 | 0.236857 |
| 45 | 6 | -5.901 | -1.74201 | -0.22329 |
| 46 | 6 | -6.44855 | -2.61363 | -1.17492 |
| 47 | 6 | -6.74272 | -1.25457 | 0.795552 |
| 48 | 6 | -7.79592 | -2.98135 | -1.12277 |
| 49 | 1 | -5.82319 | -2.99676 | -1.97528 |
| 50 | 6 | -8.07585 | -1.62796 | 0.848456 |
| 51 | 1 | -6.36105 | -0.59121 | 1.56416 |
| 52 | 6 | -8.62679 | -2.49771 | -0.10987 |
| 53 | 1 | -8.20533 | -3.65362 | -1.87262 |
| 54 | 6 | -10.2191 | -1.24941 | 1.788948 |
| 55 | 6 | -10.81 | -2.10692 | 0.845657 |
| 56 | 1 | -10.3996 | -3.32081 | -0.77731 |
| 57 | 6 | -10.9982 | -0.49734 | 2.657118 |
| 58 | 6 | -12.2036 | -2.19224 | 0.792398 |
| 59 | 6 | -12.3943 | -0.59728 | 2.604942 |
| 60 | 1 | -10.5018 | 0.155194 | 3.367811 |
| 61 | 1 | -12.6682 | -2.85445 | 0.065902 |
| 62 | 6 | -12.9934 | -1.444 | 1.672208 |
| 63 | 1 | -13.0014 | -0.0135 | 3.289094 |
| 64 | 1 | -14.0744 | -1.52725 | 1.621227 |
| 65 | 6 | -4.32605 | 1.227049 | -0.06106 |
| 66 | 6 | -3.49771 | 2.228081 | 0.47635 |
| 67 | 6 | -5.6001 | 1.600211 | -0.54196 |
| 68 | 6 | -3.93047 | 3.551381 | 0.546643 |
| 69 | 1 | -2.51137 | 1.969491 | 0.843528 |
| 70 | 6 | -6.02485 | 2.913713 | -0.45725 |
| 71 | 1 | -6.26575 | 0.874504 | -0.99278 |
| 72 | 6 | -5.20137 | 3.914399 | 0.091478 |
| 73 | 1 | -3.28017 | 4.311601 | 0.971756 |
| 74 | 6 | -7.63323 | 4.541995 | -1.09158 |
| 75 | 6 | -6.84047 | 5.569233 | -0.55182 |
| 76 | 1 | -5.04195 | 5.951047 | 0.401895 |
| 77 | 6 | -8.79138 | 4.832734 | -1.79924 |
| 78 | 6 | -7.23498 | 6.896207 | -0.74074 |
| 79 | 6 | -9.18533 | 6.165224 | -1.97583 |
| 80 | 1 | -9.37491 | 4.010341 | -2.19996 |
| 81 | 1 | -6.62661 | 7.695656 | -0.32508 |
| 82 | 6 | -8.40578 | 7.193675 | -1.44649 |
| 83 | 1 | -10.0945 | 6.389193 | -2.52416 |
| 84 | 1 | -8.7003 | 8.229922 | -1.579 |
| 85 | 7 | -5.69566 | 5.216082 | 0.174046 |
| 86 | 7 | -9.97142 | -2.86149 | 0.013584 |
| 87 | 8 | -8.83983 | -1.15539 | 1.902604 |
| 88 | 8 | -7.30085 | 3.20853 | -0.90939 |
| 89 | 6 | 12.58462 | -1.50904 | -0.30836 |
| 90 | 7 | 13.58176 | -2.09188 | -0.45404 |

## JK-POZ-3

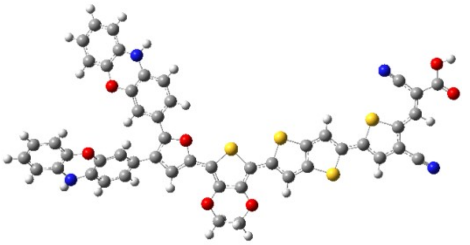

| center Number | Atomic Number | Coordinates (Angsroms) | | |
|---|---|---|---|---|
| | | X | Y | Z |
| 1 | 6 | -0.11906 | -1.5388 | -0.2951 |
| 2 | 6 | -1.22619 | -2.34233 | -0.43361 |
| 3 | 6 | -2.37654 | -1.51081 | -0.25825 |
| 4 | 1 | -1.21715 | -3.4088 | -0.59916 |
| 5 | 6 | -1.89856 | -0.22735 | -0.04002 |
| 6 | 6 | 1.289848 | -1.78254 | -0.34367 |
| 7 | 6 | 1.938194 | -2.97849 | -0.59576 |
| 8 | 6 | 3.353607 | -2.87811 | -0.56426 |
| 9 | 6 | 3.823481 | -1.59766 | -0.30309 |
| 10 | 6 | 2.140795 | -5.15542 | -1.41216 |
| 11 | 6 | 3.469252 | -5.21199 | -0.67598 |
| 12 | 1 | 1.605146 | -6.10279 | -1.32225 |
| 13 | 1 | 4.130913 | -5.95969 | -1.11803 |
| 14 | 6 | 5.186579 | -1.15874 | -0.20357 |
| 15 | 6 | 6.335075 | -1.90146 | -0.45885 |
| 16 | 6 | 7.277738 | 0.184632 | 0.157339 |
| 17 | 6 | 7.505181 | -1.14112 | -0.25303 |
| 18 | 1 | 6.302894 | -2.93262 | -0.77986 |
| 19 | 6 | 8.435821 | 0.946406 | 0.358288 |
| 20 | 6 | 9.595252 | 0.20796 | 0.101945 |
| 21 | 1 | 8.451675 | 1.981461 | 0.67308 |
| 22 | 6 | 10.96936 | 0.56549 | 0.174543 |
| 23 | 6 | 11.55699 | 1.759142 | 0.525626 |
| 24 | 6 | 13.03653 | 1.825258 | 0.506042 |
| 25 | 6 | 10.81508 | 2.914907 | 0.901235 |
| 26 | 8 | 13.7722 | 0.901948 | 0.202598 |
| 27 | 8 | 13.50693 | 3.042462 | 0.868056 |
| 28 | 1 | 14.47637 | 2.993773 | 0.827229 |
| 29 | 1 | 11.6885 | -0.20988 | -0.08318 |
| 30 | 16 | 9.202859 | -1.45795 | -0.39523 |
| 31 | 16 | 5.565674 | 0.500405 | 0.301835 |
| 32 | 16 | 2.459321 | -0.50401 | -0.08564 |
| 33 | 7 | 10.17219 | 3.839641 | 1.203056 |
| 34 | 8 | 1.284206 | -4.15649 | -0.83385 |
| 35 | 8 | 4.16393 | -3.95509 | -0.78362 |
| 36 | 1 | 2.300259 | -4.92627 | -2.47378 |
| 37 | 1 | 3.311747 | -5.44532 | 0.384493 |
| 38 | 8 | -0.52308 | -0.25221 | -0.05352 |
| 39 | 6 | -3.77119 | -2.00031 | -0.26413 |
| 40 | 6 | -4.65607 | -1.69872 | 0.785921 |
| 41 | 6 | -4.23519 | -2.8242 | -1.30102 |
| 42 | 6 | -5.95286 | -2.20921 | 0.80944 |
| 43 | 1 | -4.31931 | -1.07516 | 1.608413 |
| 44 | 6 | -5.54329 | -3.30713 | -1.30317 |
| 45 | 1 | -3.57659 | -3.06655 | -2.12962 |
| 46 | 6 | -6.41731 | -3.01284 | -0.24905 |
| 47 | 1 | -5.89174 | -3.92051 | -2.13082 |
| 48 | 6 | -8.59122 | -1.99273 | 1.457813 |
| 49 | 6 | -8.79807 | -2.81194 | 0.333406 |
| 50 | 1 | -7.97028 | -4.0612 | -1.0522 |
| 51 | 6 | -9.65931 | -1.2808 | 2.008743 |
| 52 | 6 | -10.0872 | -2.92008 | -0.20497 |
| 53 | 1 | -9.48038 | -0.63359 | 2.862234 |
| 54 | 6 | -10.9471 | -1.41144 | 1.481263 |
| 55 | 6 | -11.1579 | -2.23827 | 0.375899 |
| 56 | 1 | -10.2489 | -3.54697 | -1.0789 |
| 57 | 1 | -11.7726 | -0.86729 | 1.928619 |
| 58 | 1 | -12.1521 | -2.34511 | -0.04718 |
| 59 | 6 | -2.50084 | 1.086178 | 0.153847 |
| 60 | 6 | -1.777 | 2.117391 | 0.781568 |
| 61 | 6 | -3.79936 | 1.375079 | -0.31084 |
| 62 | 6 | -2.33158 | 3.383162 | 0.938168 |
| 63 | 1 | -0.77107 | 1.927318 | 1.138065 |
| 64 | 6 | -4.3719 | 2.628511 | -0.1141 |
| 65 | 1 | -4.37193 | 0.613424 | -0.82743 |
| 66 | 6 | -3.63646 | 3.657459 | 0.504872 |
| 67 | 1 | -1.74988 | 4.170812 | 1.411034 |
| 68 | 6 | -6.02828 | 4.650446 | -0.90305 |
| 69 | 6 | -5.13064 | 5.479146 | -0.20591 |
| 70 | 1 | -3.58408 | 5.598818 | 1.122139 |
| 71 | 6 | -6.93857 | 5.210629 | -1.80248 |
| 72 | 6 | -5.18018 | 6.864696 | -0.41007 |
| 73 | 6 | -6.99806 | 6.59519 | -1.9847 |
| 74 | 1 | -7.61225 | 4.557538 | -2.34945 |
| 75 | 6 | -6.11897 | 7.420615 | -1.28062 |
| 76 | 1 | -4.48215 | 7.506746 | 0.122181 |
| 77 | 1 | -7.72182 | 7.019772 | -2.67295 |
| 78 | 1 | -6.15052 | 8.497407 | -1.41558 |
| 79 | 7 | -7.72443 | -3.52077 | -0.23371 |
| 80 | 7 | -4.20198 | 4.924424 | 0.691153 |
| 81 | 16 | -6.08022 | 2.899164 | -0.55757 |
| 82 | 16 | -6.98686 | -1.94712 | 2.241285 |

## JK-PTZ

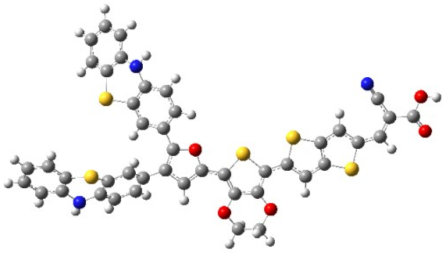

| center Number | Atomic Number | Coordinates (Angsroms) | | |
| --- | --- | --- | --- | --- |
| | | X | Y | Z |
| 1 | 6 | -1.84088 | -1.76655 | -0.27534 |
| 2 | 6 | -3.02311 | -2.46345 | -0.35702 |
| 3 | 6 | -4.08366 | -1.51643 | -0.199 |
| 4 | 1 | -3.12096 | -3.53188 | -0.47407 |
| 5 | 6 | -3.47943 | -0.27781 | -0.04816 |
| 6 | 6 | -0.46331 | -2.14901 | -0.33962 |
| 7 | 6 | 0.060516 | -3.41189 | -0.54758 |
| 8 | 6 | 1.480058 | -3.44912 | -0.54788 |
| 9 | 6 | 2.077539 | -2.21148 | -0.35635 |
| 10 | 6 | 0.032678 | -5.63415 | -1.26097 |
| 11 | 6 | 1.366739 | -5.78542 | -0.5485 |
| 12 | 1 | -0.58978 | -6.51942 | -1.11448 |
| 13 | 1 | 1.941315 | -6.61538 | -0.96495 |
| 14 | 6 | 3.480204 | -1.90404 | -0.3057 |
| 15 | 6 | 4.545709 | -2.761 | -0.5578 |
| 16 | 6 | 5.700257 | -0.75712 | -0.04697 |
| 17 | 6 | 5.787608 | -2.10673 | -0.40824 |
| 18 | 1 | 4.407771 | -3.79559 | -0.83744 |
| 19 | 6 | 6.941962 | -0.10869 | 0.093811 |
| 20 | 6 | 8.007968 | -0.96214 | -0.15981 |
| 21 | 1 | 7.067758 | 0.932792 | 0.366539 |
| 22 | 16 | 7.45048 | -2.59512 | -0.57974 |
| 23 | 16 | 4.033169 | -0.27256 | 0.125672 |
| 24 | 16 | 0.831541 | -0.98082 | -0.1658 |
| 25 | 8 | -0.70945 | -4.53044 | -0.7165 |
| 26 | 8 | 2.177249 | -4.61001 | -0.73292 |
| 27 | 1 | 0.188536 | -5.47208 | -2.33547 |
| 28 | 1 | 1.212298 | -5.95109 | 0.525191 |
| 29 | 8 | -2.11301 | -0.43736 | -0.08543 |
| 30 | 6 | 9.411557 | -0.66398 | -0.11551 |
| 31 | 6 | 10.47612 | -1.52278 | -0.37815 |
| 32 | 16 | 9.972523 | 0.942705 | 0.30837 |
| 33 | 6 | 11.70421 | -0.87374 | -0.23392 |
| 34 | 1 | 10.37937 | -2.56334 | -0.66005 |
| 35 | 6 | 11.66409 | 0.470582 | 0.133809 |
| 36 | 6 | 12.80993 | 1.280979 | 0.323911 |
| 37 | 6 | 12.92192 | 2.602677 | 0.681222 |
| 38 | 6 | 11.79779 | 3.439472 | 0.93969 |
| 39 | 6 | 14.28728 | 3.16794 | 0.798438 |
| 40 | 7 | 10.86362 | 4.105766 | 1.146549 |
| 41 | 1 | 15.20726 | 4.757764 | 1.207175 |
| 42 | 8 | 15.31653 | 2.546825 | 0.601126 |
| 43 | 8 | 14.27956 | 4.473905 | 1.154832 |
| 44 | 1 | 13.75898 | 0.774518 | 0.160247 |
| 45 | 6 | -5.51884 | -1.86761 | -0.15742 |
| 46 | 6 | -6.08324 | -2.6862 | -1.1477 |
| 47 | 6 | -6.34665 | -1.43726 | 0.894292 |
| 48 | 6 | -7.43129 | -3.04003 | -1.10397 |
| 49 | 1 | -5.46979 | -3.02635 | -1.97657 |
| 50 | 6 | -7.6855 | -1.8187 | 0.964792 |
| 51 | 1 | -5.9329 | -0.81483 | 1.68177 |
| 52 | 6 | -8.24872 | -2.61821 | -0.0478 |
| 53 | 1 | -7.85574 | -3.65162 | -1.89674 |
| 54 | 6 | -10.2757 | -1.32322 | 1.653614 |
| 55 | 6 | -10.5856 | -2.16547 | 0.570626 |
| 56 | 1 | -9.91267 | -3.54514 | -0.77442 |
| 57 | 6 | -11.2585 | -0.49021 | 2.193461 |
| 58 | 6 | -11.8915 | -2.17243 | 0.062729 |
| 59 | 6 | -12.565 | -0.51934 | 1.697493 |
| 60 | 1 | -10.9991 | 0.172086 | 3.01408 |
| 61 | 1 | -12.1323 | -2.81711 | -0.77948 |
| 62 | 6 | -12.879 | -1.36782 | 0.633803 |
| 63 | 1 | -13.3247 | 0.119354 | 2.136527 |
| 64 | 1 | -13.8885 | -1.39689 | 0.235265 |
| 65 | 6 | -3.94783 | 1.095481 | 0.095862 |
| 66 | 6 | -3.11591 | 2.077684 | 0.665451 |
| 67 | 6 | -5.22113 | 1.48896 | -0.36158 |
| 68 | 6 | -3.54282 | 3.397065 | 0.773432 |
| 69 | 1 | -2.1262 | 1.806183 | 1.014636 |
| 70 | 6 | -5.66627 | 2.799473 | -0.21258 |
| 71 | 1 | -5.87428 | 0.764952 | -0.83491 |
| 72 | 6 | -4.82335 | 3.777806 | 0.348381 |
| 73 | 1 | -2.87881 | 4.144249 | 1.201502 |
| 74 | 6 | -7.13292 | 4.936879 | -1.0693 |
| 75 | 6 | -6.14657 | 5.703726 | -0.42302 |
| 76 | 1 | -4.5727 | 5.729823 | 0.877493 |
| 77 | 6 | -8.00032 | 5.543143 | -1.98119 |
| 78 | 6 | -6.06467 | 7.077027 | -0.68999 |
| 79 | 6 | -7.92807 | 6.91741 | -2.22595 |
| 80 | 1 | -8.74397 | 4.935546 | -2.48849 |
| 81 | 1 | -5.29808 | 7.670559 | -0.1972 |

| center Number | Atomic Number | Coordinates (Angsroms) | | |
| --- | --- | --- | --- | --- |
| | | X | Y | Z |
| 82 | 6 | -6.96034 | 7.683111 | -1.57244 |
| 83 | 1 | -8.61926 | 7.379958 | -2.92317 |
| 84 | 1 | -6.88938 | 8.750755 | -1.75631 |
| 85 | 7 | -5.26099 | 5.100805 | 0.486408 |
| 86 | 7 | -9.59768 | -2.99703 | 0.014722 |
| 87 | 9 | 12.87031 | -1.51193 | -0.43942 |
| 88 | 16 | -7.34841 | 3.21609 | -0.6433 |
| 89 | 16 | -8.65633 | -1.39811 | 2.403127 |

# JK-PTZ-1

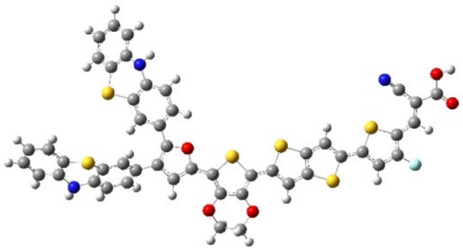

| center Number | Atomic Number | Coordinates (Angsroms) | | |
|---|---|---|---|---|
| | | X | Y | Z |
| 1 | 6 | -2.44265 | -1.72309 | -0.25218 |
| 2 | 6 | -3.61038 | -2.44388 | -0.33675 |
| 3 | 6 | -4.69038 | -1.51762 | -0.18922 |
| 4 | 1 | -3.68626 | -3.5146 | -0.44908 |
| 5 | 6 | -4.11179 | -0.26632 | -0.0413 |
| 6 | 6 | -1.05764 | -2.07838 | -0.30704 |
| 7 | 6 | -0.50789 | -3.33195 | -0.50545 |
| 8 | 6 | 0.911877 | -3.34112 | -0.49815 |
| 9 | 6 | 1.483904 | -2.09065 | -0.3103 |
| 10 | 6 | -0.48809 | -5.55784 | -1.20765 |
| 11 | 6 | 0.844818 | -5.67927 | -0.48741 |
| 12 | 1 | -1.09378 | -6.45443 | -1.05991 |
| 13 | 1 | 1.438098 | -6.49944 | -0.89691 |
| 14 | 6 | 2.879634 | -1.75572 | -0.25435 |
| 15 | 6 | 3.963132 | -2.59423 | -0.49267 |
| 16 | 6 | 5.075566 | -0.56382 | 0.006643 |
| 17 | 6 | 5.191027 | -1.91488 | -0.34205 |
| 18 | 1 | 3.84682 | -3.63374 | -0.76369 |
| 19 | 6 | 6.303791 | 0.109212 | 0.149002 |
| 20 | 6 | 7.387439 | -0.72613 | -0.09068 |
| 21 | 1 | 6.408106 | 1.155232 | 0.413384 |
| 22 | 16 | 6.863719 | -2.3732 | -0.49945 |
| 23 | 16 | 3.398578 | -0.10986 | 0.165235 |
| 24 | 16 | 0.212812 | -0.88384 | -0.13249 |
| 25 | 8 | -1.25474 | -4.46618 | -0.67262 |
| 26 | 8 | 1.632844 | -4.48867 | -0.67301 |
| 27 | 1 | -0.32982 | -5.39799 | -2.2821 |
| 28 | 1 | 0.688064 | -5.84292 | 0.586219 |
| 29 | 8 | -2.74232 | -0.39867 | -0.07035 |
| 30 | 6 | 8.784983 | -0.40132 | -0.04082 |
| 31 | 6 | 9.869767 | -1.24119 | -0.28663 |
| 32 | 16 | 9.32833 | 1.207248 | 0.364916 |
| 33 | 6 | 11.10833 | -0.59823 | -0.15023 |
| 34 | 1 | 9.768404 | -2.28433 | -0.55605 |
| 35 | 6 | 11.02093 | 0.756462 | 0.204787 |
| 36 | 6 | 12.12032 | 1.63874 | 0.407229 |
| 37 | 6 | 12.14826 | 2.966716 | 0.752801 |
| 38 | 6 | 10.98302 | 3.752809 | 0.987027 |
| 39 | 6 | 13.48254 | 3.608289 | 0.886693 |
| 40 | 7 | 10.0219 | 4.384918 | 1.175975 |
| 41 | 1 | 14.30438 | 5.251544 | 1.294752 |
| 42 | 8 | 14.54455 | 3.040475 | 0.711934 |
| 43 | 8 | 13.39563 | 4.913693 | 1.229415 |
| 44 | 1 | 13.10622 | 1.20312 | 0.268927 |
| 45 | 6 | -6.11845 | -1.89736 | -0.1539 |
| 46 | 6 | -6.66106 | -2.73034 | -1.14434 |
| 47 | 6 | -6.9602 | -1.48033 | 0.892103 |
| 48 | 6 | -8.00193 | -3.11104 | -1.10639 |
| 49 | 1 | -6.03658 | -3.06082 | -1.96888 |
| 50 | 6 | -8.29144 | -1.88842 | 0.957019 |
| 51 | 1 | -6.56318 | -0.84709 | 1.679597 |
| 52 | 6 | -8.8332 | -2.70233 | -0.05586 |
| 53 | 1 | -8.40983 | -3.73362 | -1.89928 |
| 54 | 6 | -10.8946 | -1.44348 | 1.631358 |
| 55 | 6 | -11.1819 | -2.29511 | 0.549488 |
| 56 | 1 | -10.4744 | -3.66481 | -0.78805 |
| 57 | 6 | -11.8969 | -0.62909 | 2.163816 |
| 58 | 6 | -12.4849 | -2.33024 | 0.03524 |
| 59 | 6 | -13.2 | -0.68633 | 1.661543 |
| 60 | 1 | -11.6551 | 0.040822 | 2.983648 |
| 61 | 1 | -12.7082 | -2.98223 | -0.80613 |
| 62 | 6 | -13.4914 | -1.54423 | 0.598974 |
| 63 | 1 | -13.9747 | -0.06197 | 2.094904 |
| 64 | 1 | -14.4981 | -1.59512 | 0.195594 |
| 65 | 6 | -4.60837 | 1.09789 | 0.093401 |
| 66 | 6 | -3.79843 | 2.099965 | 0.660093 |
| 67 | 6 | -5.88798 | 1.462865 | -0.37019 |
| 68 | 6 | -4.25238 | 3.410945 | 0.759321 |
| 69 | 1 | -2.80458 | 1.850616 | 1.013992 |
| 70 | 6 | -6.36011 | 2.764829 | -0.22998 |
| 71 | 1 | -6.52483 | 0.723107 | -0.84135 |
| 72 | 6 | -5.539 | 3.763246 | 0.328063 |
| 73 | 1 | -3.6051 | 4.173823 | 1.185324 |
| 74 | 6 | -7.86763 | 4.867033 | -1.10259 |
| 75 | 6 | -6.89903 | 5.657419 | -0.45787 |
| 76 | 1 | -5.32973 | 5.722855 | 0.846892 |
| 77 | 6 | -8.74467 | 5.450454 | -2.02014 |
| 78 | 6 | -6.84448 | 7.03063 | -0.73211 |
| 79 | 6 | -8.69985 | 6.824567 | -2.27223 |
| 80 | 1 | -9.47433 | 4.825056 | -2.52613 |
| 81 | 1 | -6.09159 | 7.642398 | -0.24049 |
| 82 | 6 | -7.74984 | 7.613457 | -1.62031 |
| 83 | 1 | -9.39839 | 7.269057 | -2.97384 |
| 84 | 1 | -7.70022 | 8.681319 | -1.80981 |
| 85 | 7 | -6.00374 | 5.077727 | 0.457173 |
| 86 | 7 | -10.1745 | -3.10794 | 0.000854 |
| 87 | 6 | 12.40987 | -1.32045 | -0.37098 |
| 88 | 9 | 13.14235 | -0.76292 | -1.36875 |
| 89 | 9 | 13.1944 | -1.31261 | 0.736911 |
| 90 | 9 | 12.20823 | -2.61667 | -0.70433 |
| 91 | 16 | -9.27782 | -1.48312 | 2.389099 |
| 92 | 16 | -8.04912 | 3.144565 | -0.66769 |

## JK-PTZ-2

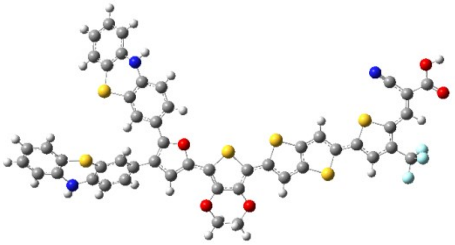

| center Number | Atomic Number | Coordinates (Angsroms) X | Y | Z |
|---|---|---|---|---|
| 1 | 6 | -1.94712 | -1.75544 | -0.26789 |
| 2 | 6 | -3.12532 | -2.4593 | -0.35098 |
| 3 | 6 | -4.19147 | -1.51845 | -0.19666 |
| 4 | 1 | -3.21676 | -3.52845 | -0.46674 |
| 5 | 6 | -3.59455 | -0.27592 | -0.0464 |
| 6 | 6 | -0.56769 | -2.13027 | -0.32877 |
| 7 | 6 | -0.03674 | -3.39097 | -0.53414 |
| 8 | 6 | 1.382608 | -3.42056 | -0.5319 |
| 9 | 6 | 1.973212 | -2.17896 | -0.3412 |
| 10 | 6 | -0.0515 | -5.61328 | -1.24714 |
| 11 | 6 | 1.281937 | -5.75777 | -0.53221 |
| 12 | 1 | -0.66952 | -6.50181 | -1.10192 |
| 13 | 1 | 1.862183 | -6.58388 | -0.94834 |
| 14 | 6 | 3.373548 | -1.86461 | -0.28895 |
| 15 | 6 | 4.444111 | -2.7188 | -0.53138 |
| 16 | 6 | 5.587214 | -0.7051 | -0.03328 |
| 17 | 6 | 5.68191 | -2.05748 | -0.38373 |
| 18 | 1 | 4.311836 | -3.75622 | -0.80298 |
| 19 | 6 | 6.825525 | -0.05051 | 0.106065 |
| 20 | 6 | 7.895892 | -0.9016 | -0.13767 |
| 21 | 1 | 6.945535 | 0.993699 | 0.371055 |
| 22 | 16 | 7.347174 | -2.54025 | -0.54642 |
| 23 | 16 | 3.91753 | -0.22685 | 0.130524 |
| 24 | 16 | 0.720176 | -0.95474 | -0.15375 |
| 25 | 8 | -0.80041 | -4.51348 | -0.70357 |
| 26 | 8 | 2.086412 | -4.57731 | -0.71375 |
| 27 | 1 | 0.105226 | -5.44998 | -2.32128 |
| 28 | 1 | 1.126631 | -5.92509 | 0.541023 |
| 29 | 8 | -2.22728 | -0.42751 | -0.08045 |
| 30 | 6 | 9.298273 | -0.59697 | -0.09161 |
| 31 | 6 | 10.36954 | -1.44765 | -0.34265 |
| 32 | 16 | 9.861089 | 1.009641 | 0.319161 |
| 33 | 6 | 11.62173 | -0.81447 | -0.20654 |
| 34 | 1 | 10.26156 | -2.48995 | -0.61566 |
| 35 | 6 | 11.54448 | 0.544037 | 0.153926 |
| 36 | 6 | 12.66982 | 1.391114 | 0.349486 |
| 37 | 6 | 12.73759 | 2.716471 | 0.697646 |
| 38 | 6 | 11.59091 | 3.526938 | 0.94021 |
| 39 | 6 | 14.08846 | 3.324902 | 0.824542 |
| 40 | 7 | 10.6411 | 4.174241 | 1.134782 |
| 41 | 1 | 14.95139 | 4.947685 | 1.231517 |
| 42 | 8 | 15.13363 | 2.730412 | 0.641417 |
| 43 | 8 | 14.03469 | 4.630913 | 1.170767 |
| 44 | 1 | 13.63779 | 0.915531 | 0.199795 |
| 45 | 6 | -5.62471 | -1.87781 | -0.15758 |
| 46 | 6 | -6.18263 | -2.69973 | -1.14876 |
| 47 | 6 | -6.45671 | -1.45203 | 0.892672 |
| 48 | 6 | -7.5287 | -3.06123 | -1.10728 |
| 49 | 1 | -5.56588 | -3.03648 | -1.97657 |
| 50 | 6 | -7.79345 | -1.84115 | 0.961021 |
| 51 | 1 | -6.04791 | -0.82714 | 1.680803 |
| 52 | 6 | -8.35036 | -2.64397 | -0.05253 |
| 53 | 1 | -7.94826 | -3.67534 | -1.90067 |
| 54 | 6 | -10.3876 | -1.36134 | 1.6462 |
| 55 | 6 | -10.6908 | -2.20522 | 0.56265 |
| 56 | 1 | -10.0077 | -3.58065 | -0.78177 |
| 57 | 6 | -11.3762 | -0.53455 | 2.184969 |
| 58 | 6 | -11.996 | -2.22015 | 0.052997 |
| 59 | 6 | -12.6818 | -0.57162 | 1.687228 |
| 60 | 1 | -11.122 | 0.129098 | 3.006132 |
| 61 | 1 | -12.2316 | -2.86613 | -0.78965 |
| 62 | 6 | -12.9892 | -1.42177 | 0.622931 |
| 63 | 1 | -13.446 | 0.062213 | 2.125431 |
| 64 | 1 | -13.9979 | -1.45701 | 0.223072 |
| 65 | 6 | -4.07153 | 1.094585 | 0.094493 |
| 66 | 6 | -3.24639 | 2.083041 | 0.663178 |
| 67 | 6 | -5.34684 | 1.479124 | -0.36509 |
| 68 | 6 | -3.6817 | 3.399848 | 0.768186 |
| 69 | 1 | -2.25546 | 1.818419 | 1.014182 |
| 70 | 6 | -5.80038 | 2.787042 | -0.21924 |
| 71 | 1 | -5.99497 | 0.750063 | -0.83755 |
| 72 | 6 | -4.96412 | 3.771783 | 0.340776 |
| 73 | 1 | -3.02301 | 4.151997 | 1.195753 |
| 74 | 6 | -7.28048 | 4.913408 | -1.08031 |
| 75 | 6 | -6.29953 | 5.687724 | -0.43484 |
| 76 | 1 | -4.72635 | 5.726278 | 0.866481 |
| 77 | 6 | -8.15155 | 5.512556 | -1.99341 |
| 78 | 6 | -6.22658 | 7.061125 | -0.70371 |
| 79 | 6 | -8.08825 | 6.886898 | -2.24015 |
| 80 | 1 | -8.891 | 4.899343 | -2.50007 |
| 81 | 1 | -5.46416 | 7.660444 | -0.21146 |

| center Number | Atomic Number | Coordinates (Angsroms) X | Y | Z |
|---|---|---|---|---|
| 82 | 6 | -7.12588 | 7.659966 | -1.5874 |
| 83 | 1 | -8.78223 | 7.343843 | -2.93827 |
| 84 | 1 | -7.06197 | 8.727783 | -1.77279 |
| 85 | 7 | -5.40998 | 5.092055 | 0.475646 |
| 86 | 7 | -9.69705 | -3.03047 | 0.007662 |
| 87 | 6 | 12.85789 | -1.49686 | -0.41752 |
| 88 | 7 | 13.85783 | -2.06659 | -0.59281 |
| 89 | 16 | -7.48482 | 3.191854 | -0.6519 |
| 90 | 16 | -8.76877 | -1.42637 | 2.397923 |

## JK-PTZ-3

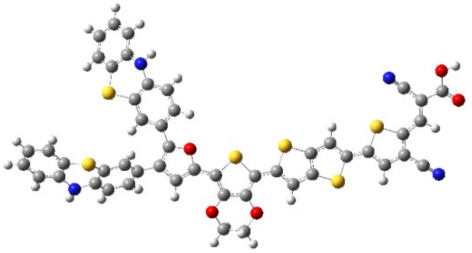

Supplement: RA-012-D2RA00906D-s001 [file RA-012-D2RA00906D-s001.pdf]
